# Polystyrene Nanoplastics Exacerbate HFD-induced MASLD by Reducing Cathepsin Activity and Triggering Large Vacuole Formation via Impaired Lysosomal Acidification

**DOI:** 10.7150/ijbs.108268

**Published:** 2025-06-09

**Authors:** Jiwon Ahn, Kajung Ryu, Hyerin Kim, Hwi Won Seo, Minsu Jang, Seung-Hyun Kim, Yunho Park, Myung Jin Son, Ho-Joon Lee, Ok-Seon Kwon, Kyung-Sook Chung

**Affiliations:** 1 Center for Gene and Cell Therapy, Korea Research Institute of Bioscience and Biotechnology (KRIBB), 125 Gwahak-ro, Yuseong-gu, Daejeon 34141, Republic of Korea.; 2 Korea Bioinformation Center, Korea Research Institute of Bioscience and Biotechnology (KRIBB), 125 Gwahak-ro, Yuseong-gu, Daejeon 34141, Republic of Korea.; 3 Infectious Diseases Research Center, Korea Research Institute of Bioscience and Biotechnology (KRIBB), 125 Gwahak-ro, Yuseong-gu, Daejeon 34141, Republic of Korea.; 4 Advanced Bioconvergence Department, Korea University of Science and Technology (UST), Daejeon 34141, Republic of Korea.; 5 Biomedical Translational Research Center, Korea Research Institute of Bioscience and Biotechnology (KRIBB), 125 Gwahak-ro, Yuseong-gu, Daejeon 34141, Republic of Korea.

**Keywords:** polystyrene nanoparticle, high-fat diet, impaired lysosome acidification, large vacuoles, cathepsins

## Abstract

Environmental nanoplastics (NPs) have harmful effects on health. This study investigated the effects of polystyrene (PS) NPs on steatosis and fatty liver disease. PS-NP oral administration, in conjunction with a high-fat diet (HFD), synergistically exacerbated the symptoms of steatosis in mice, leading to increased alanine transaminase, aspartate aminotransferase, and cholesterol levels; no effects were observed with PS-NPs on a normal chow diet. Transcriptome analysis unveiled that PS-NPs interfered with actin organization, cell-cell adhesion, PPAR signaling, and lipid metabolism. In HepaRG cells, PS-NPs rapidly entered by inducing actin rearrangement, resulting in the formation of numerous small cytoplasmic vesicles. This treatment led to an augmented number of acidic organelles, leading to development and buildup of large vacuoles, indicative of enlarged pre-lysosomal and lysosomal compartments. PS-NP exposure hampered p62 degradation, leading to LC3B accumulation and decreased cathepsin B and D activity. Additionally, PS-NP exposure resulted in accumulation of lipid droplets and elevated expression of lipogenesis-, transport-, and storage-related genes. These findings suggest that excessive endocytosis driven by PS-NPs worsens MASLD in HFD through accumulation of lysosomes and large vacuoles with reduced cathepsin activity.

## Introduction

The emergence of microplastics (MPs) and nanoplastics (NPs) has raised concerns owing to their potential implications for both environmental and human health. Their small size allows them to infiltrate tissues and biological barriers, thereby posing a significant risk of toxicity. Increased cellular absorption of NPs has been linked to various health issues, including genotoxicity, inflammation, immune dysfunction, neurotoxicity, metabolic changes, and carcinogenicity. Ingested MPs/NPs can travel from the gut to various organs in marine organisms and mammals 1-4. The liver, which has essential roles in digestion, absorption, and detoxification, is intricately connected to the gastrointestinal tract through the hepatic portal vein and often accumulates high levels of ingested MPs/NPs 5, 6.

Exposure to MP/NPs was found to disrupt hepatic glycolipid metabolism in aquatic fish and to cause organ injury and abnormal lipid metabolism in mice 2, 3, 7, 8. Biochemical and metabolomics analyses of mouse livers following exposure to MP/NPs revealed altered oxidative stress, energy metabolism, lipid metabolism, and neurotoxicity 9, 10. Induced metabolic disruptions, particularly in relation to lipids, may indirectly contribute to the development of metabolic dysfunction-associated steatotic liver disease (MASLD). Furthermore, studies have associated exposure to dietary NPs in mice fed a normal chow diet (CD) or a high-fat diet (HFD) with insulin resistance and elevated levels of plasma lipopolysaccharide and pro-inflammatory cytokines 11. Other NPs, including silica nanoparticles and polystyrene nanoplastics (PS-NPs), has been found to induce oxidative damage and pro-inflammatory responses in hepatic tissue, potentially exacerbating fatty liver disease in HFD-fed mice 12, 13. In mice, 10 days of exposure to 250 nm polyurethane nanoparticles, administered via oral gavage, resulted in elevated serum alanine aminotransferase (ALT), alkaline phosphatase, interleukin (IL)-6, and tumor necrosis factor-α levels, accompanied by liver vascular congestion and hepatocyte vacuolization 14, 15. However, no significant changes in hepatic function were found following exposure to 5 µm PS microparticles (20 mg/kg/day body weight) in drinking water for 30 days 16. Vacuolization of endosomal-lysosomal components in the liver may result from organelle sorting, fusion disruption, or changes in the intraorganellar ionic balance, impairing macropinocytosis, endocytosis, and autophagy. Moreover, few studies have investigated the potential mechanistic link between NP exposure and the development of chronic liver disease in obese patients with MASLD 12, 13, 17. Nevertheless, previous studies using pure MPs/NPs have not reported serious cytotoxic effects on human cells 18-20, and the effects of dietary NP exposure on mammalian cells and tissues, particularly in humans, remain largely unexplored.

Autophagy, the process of breaking down and recycling cellular components such as lipids, is indispensable for the proper functioning of various organs, especially the liver. It is a critical driver of cellular homeostasis and plays a role in the adaptive response of cells to stress 21-23. Any aberration in autophagy can lead to detrimental effects, as evidenced by prior studies showing that exposure to metal-based nanoparticles, Fe_3_O_4_-nanoparticles, resulted in autophagosome accumulation in treated cells 24. Similarly, exposure to NPs has been linked to impaired autophagy in liver cells, resulting in lipid accumulation and the onset of fatty liver disease. These effects are thought to be resulted from lysosomal defects induced by NP exposure, disrupting cellular waste breakdown, homeostasis maintenance, and impacting membrane traffic, repair, metabolism, and signaling processes 6. Recent findings have shown that NPs were predominantly accumulated in the lysosomes of earthworm cells, irrespective of their surface charge 25. Similarly, in zebrafish liver cells, 65 nm PS-NPs were internalized and accumulated in lysosomes, resulting in altered pH and membrane integrity, and lysosomal dysfunction 6. Impaired lysosomal function can also lead to lipid accumulation in cells, since lysosomes play a pivotal role in regulating lipid catabolism. Although the precise mechanisms underlying NP-induced autophagy impairment are unclear, plausible explanations include interference with lysosomal function and the disruption of autophagic flux, the process by which cellular components are transported to the lysosomes for degradation 24, 26.

In this study, we aimed to explore the combined effects of obesity and NPs on human health by the concurrent administration of a HFD and PS-NPs to mice and human liver cell lines. Our findings suggest that excessive absorption and accumulation of PS-NPs in lysosomes alter lysosomal function and integrity. Lysosomal dysfunction, in turn, leads to the accumulation of enlarged pre-lysosomal and autophagic vacuoles due to inhibited maturation of lysosomal protease cathepsins. These detrimental effects disrupt autophagy, impair lipolysis, and induce the accumulation of excess fat in the liver, ultimately contributing to MASLD development.

## Materials and Methods

### Cell culture

HepaRG (HPRGC10; Gibco, CA, USA) cells were cultured in William's E medium (Sigma-Aldrich, St. Louis, MO, USA) supplemented with 1% Glutamax (Gibco), 10% (v/v) fetal bovine serum (FBS; Welgene, Daegu, Republic of Korea), 1% (v/v) penicillin-streptomycin (Pen-Strep; Gibco), 5 ng/mL insulin (Corning, CA, USA), and 50 μM hydrocortisone hemisuccinate (Santa Cruz Biotechnology, Santa Cruz, CA, USA). HepG2 (HB-8065; ATCC, Manassas, VA, USA), Hep-G2/2.2.15 (SCC249; Sigma-Aldrich), Hep3B (88064; KCLB; Korean Cell Line Bank, Seoul, Republic of Korea), Huh7 (60104; KCLB), SK-HEP-1 (HTB-52; ATCC), 293T (CRL-3216; ATCC), and PLC/PRF/5 (CRL-8024; ATCC) cells were cultured in Dulbecco's Modified Eagle Medium (Gibco) supplemented with 10% FBS and 1% Pen-Strep. All cell cultures were maintained at 37 ºC in a 5% CO_2_ humidified incubator.

### Chemicals, reagents, and antibodies

Carboxyl-modified polystyrenes (PS-COOH NPs) with a size of 50 nm (2.5% w/v aqueous suspensions including 3.64 × 10^14^ particles/mL) were purchased from Polysciences, Inc. (Warrington, PA, USA). Bafilomycin A1 was purchased from InvivoGen (San Diego, CA, USA). Rapamycin, 3-methyladenine, cytochalasin B, 5-(N-ethyl-N-isopropyl) amiloride 27, chlorpromazine hydrochloride, genistein 23, wortmannin, sodium oleate (oleate), Triton X-100, and IGEPAL^®^ CA-630 were purchased from Sigma (St. Louis, MO, USA). LysoTracker, MitoTracker, ER-Tracker, LysoSensor^TM^ Yellow/Blue DND-160, fluorescein-conjugated dextran (40,000 MW, anionic, lysine fixable), and Hoechst 33258 were purchased from Thermo Fisher Scientific (Waltham, MA, USA). Fatty acid-free BSA was purchased from Millipore. The reagents and chemicals used are listed in Table S1. Antibodies against p62, lysosomal-associated membrane protein 1 (LAMP1), LC3A/B, early endosome antigen 1 (EEA1), RAB7, S6K, phospho-p70S6K, peroxisome proliferator-activated receptor γ (PPARγ), mTOR, phospho-mTor, TFEB, phospho-TFEB, and F4/80 were obtained from Cell Signaling Technology (Danvers, MA, USA). Antibodies against myeloperoxidase (MPO) and neutrophil were obtained from Abcam. Antibodies against LC3B, C/EBPα, C/EBPβ, LAMP2, LGP85, and PPARα were obtained from Santa Cruz Biotechnology. Secondary antibodies against mouse, rat, or rabbit IgG were either obtained from AbFrontier (Daejeon, Republic of Korea) or Invitrogen (Carlsbad, CA, USA). The primary and secondary antibodies used are listed specifically in Table S2 and S3.

### Animal studies and experimental design

PS-NPs were administered to C57BL/6J male mice (DBL, Eumseoung-gun, Chungcheongbuk-do, Republic of Korea) using PS-COOH NPs of either 50 nm. Oral administration involved the use of PS-NP-jelly cubes prepared as previously described 28, containing either 0 or 0.5 mg PS-NPs with 2.5% agarose. Following a 2-week acclimation period, 4-week-old mice were grouped as follows: i) control diet (CD, n=6, Teklad Global 18% protein rodent diet; Teklad 2018S; Envigo, Madison, WI, USA); ii) control diet with PS-NPs (0.5 mg) per day (CD + PS-NPs, n=6); iii) HFD (n=6) with 45 kcal% fat (D12451; Research Diet, New Brunswick, NJ, USA), or iv) HFD with PS-NPs (HFD + PS-NPs, n=6) for 8 or 20 weeks. To ensure that the PS-NPs were dosed accurately, mice were individually housed and weighed before and after the study. Perfused liver weights were noted under anesthesia with isoflurane, and liver-to-body weight ratios were calculated. Serum samples for triglycerides (TG, Beckman-Coulter, USA), alanine aminotransferase (ALT), aspartate aminotransferase (AST), and cholesterol (CHO) were collected 1 week before euthanization. The contents were spectrophotometrically determined based on the specific wavelengths as follows: TG, 660/800 nm; ALT and AST, 340 nm; and CHO, 540/600 nm. All experimental animal procedures adhered to the relevant ethical guidelines and were approved by the Institutional Animal Care and Use Committee of the Korea Research Institute of Bioscience & Biotechnology (KRIBB) (approval number: KRIBB-AEC-21279).

### Histochemical and histopathological evaluation

For hematoxylin and eosin (H&E) and Picro-Sirius Red staining, liver tissues were fixed for 24 h in 10% neutral buffered formalin solution (Sigma-Aldrich), embedded in paraffin, sectioned at 4 μm thickness, deparaffinized, and then stained with either hematoxylin (Agilent Technology, Santa Clara, CA, USA) and alcoholic Eosin Y (Thermo Fisher Scientific) or Picro-Sirius Red solution (Abcam) following manufacturer's instructions. Representative images were captured using a microscope (BX1; Olympus). To determine lipid deposition, liver tissues embedded in Tissue-Tek^®^ O.C.T. compound (Sakura Finetek USA, Torrance, CA, USA) and frozen in liquid N_2_ were sectioned at 10 μm using a Cryostat (Leica Microsystems, Germany). Sections were then air-dried, fixed in 10% neutral buffered formalin, rinsed in distilled water, and stained with Oil Red O (Sigma-Aldrich) solution. Representative images were captured using a Cytation 5 Live-Cell Imaging System (Biotek, Agilent Technologies). For immunofluorescence staining, 10 μm thickness frozen liver sections were blocked with 5% BSA blocking buffer containing 0.3% Triton X-100 for 1h at room temperature (RT), and incubated with primary antibodies for overnight at 4℃. After that, the sections were incubated with secondary antibodies and Hoechst 33258 for 1 h at RT in the dark. Representative images were captured using confocal microscope (ZEISS LSM 800; Carl Zeiss NTS Ltd.). Three spots from three lobes in each tissue section were randomly chosen to assess the relative area of fatty changes, and the ratio of the area of fatty changes to the whole tissue was calculated using ImageJ software (National Institutes of Health, Bethesda, MD, USA).

### Total RNA extraction, real-time PCR, and transcriptome analysis

Total RNA from HepaRG was extracted using TRIzol^®^ Reagent (Invitrogen), and mouse liver RNA was isolated using RNAlater^TM^ Solution (Invitrogen) and RNeasy mini kit (Qiagen, Valencia, CA, USA). cDNA was synthesized using the TOPscript^TM^ RT Dry Mix Kit (dT18 plus; Enzynomics, Daejeon, Republic of Korea), and real-time PCR was performed using PowerUP^TM^ SYBR^TM^ Green Master Mix (Applied Biosystems, Foster City, CA, USA). Gene transcripts were quantified using the 2^-ΔΔCT^ method 29, with *RPL13A* as the endogenous reference gene. mRNA was sequenced by ebiogen Inc. (Seoul, Republic of Korea). Detailed information on the methods is provided in Table S4. Differentially expressed gene sets were then used for over-representation analysis with the ClusterProfiler 4.0 package in R, using parameters set as adjusted *p* < 0.05 (Benjamini-Hochberg method). The biological functionality of the gene sets was investigated through Gene Ontology and Kyoto Encyclopedia of Genes and Genomes (KEGG) analyses.

### Crystal violet staining

To visualize the vacuole-like structures induced by PS-NPs, the cells were stained with crystal violet. Cells exposed to either 0, 50, or 100 μg/mL PS-NPs for 24 h were rinsed with ice-cold phosphate-buffered saline (PBS), fixed with ice-cold methanol for 10 min (Merck), and stained with 0.5% crystal violet solution (Sigma) in 25% methanol for another 10 min. After washing with distilled water, images were captured using Cytation 5 (Biotek, Agilent Technologies) and a Leica microscope.

### Assessment of neutral lipid with BODIPY and Nile Red

Intracellular lipids were detected using BODIPY (BODIPY^TM^ 493/503, Invitrogen) and Nile Red (Invitrogen), as previously reported with modifications 30. Cells exposed to different concentrations of PS-NPs (0, 10, 20, 50, and 100 μg/mL) for 24 or 48 h were fixed with 4% formaldehyde (Biosesang, Gyeonggi-do, Republic of Korea), stained with 5 μM BODIPY™ 493/503 (Invitrogen) or 5 μg/mL Nile Red for 20 min, and washed with PBS. To explore the synergistic effects between PS-NPs and oleate (Sigma-Aldrich), HepaRG cells were pre-treated with 0.2 mM oleate for 3 h and then treated with PS-NPs for 24 h, followed by BODIPY or Nile Red. Representative images were captured using a BioTek Cytation 5 Live-Cell Imaging Station (Agilent Technologies). To quantify intercellular neutral lipids using labeled BODIPY, the mean fluorescence intensity was measured by flow cytometry. Cells exposed to 0 or 100 μg/mL PS-NPs for 24 h were washed with PBS and incubated with 5 μM BODIPY 493/503 in the dark for 20 min. Cells were collected using 0.05% trypsin-EDTA, resuspended in PBS with 2% FBS, and analyzed using a flow cytometer (FACSCalibur, BD, CA, USA) and Flow Software 2.

### Determination of CYP3A4 and cathepsin B activity

CYP3A4 activity was measured using the P450-Glo^TM^ CYP3A4 assay (Promega) according to the manufacturer's instructions. Briefly, HepaRG cells were treated with various concentrations of PS-NPs for 24 h, washed, and then incubated with 1 μΜ of luciferin for 1 h. Luminescence was quantified using a GloMax Navigator luminometer (Promega, Charbonnières, France) and normalized by adenosine triphosphate (ATP) levels, determined by the CellTiter-Glo® 3D Cell Viability Assay kit (Promega). Cathepsin B activity was measured using a fluorometric cathepsin B activity assay kit from Abcam (Cambridge, UK). Briefly, cells were harvested, rinsed in cold PBS, and homogenized using a refrigerated ultra-sonicator (Bioruptor®; Diagenode, Liège, Belgium). Fluorescence intensity was recorded using a multi-mode microplate reader (SpectraMax i3x, Molecular Devices) with excitation/emission wavelengths of 400/505 nm. Data were normalized to the protein concentrations.

### Triglyceride quantification assay

Intracellular TG levels were determined using a triglyceride assay kit (Abcam), according to the manufacturer's instructions. Following 24 h exposure to PS-NPs, HepaRG cells were harvested, washed with cold PBS, lysed with 5% NP-40 (IGEPAL^®^ CA-630), and homogenized using Bioruptor® (Diagenode). TG levels in the lysates were assessed by calculating the optical density at a wavelength of 570 nm using a microplate reader (VERSAmax; Molecular Devices) and normalized to the total protein concentration.

### Labeling with organelle-specific tracers and uptake

HepaRG cells were seeded in 8- or 96-well plates. The next day, the cell culture medium was replaced with 0 or 50 μg/mL PS-NPs. After 24 h, PS-NPs were removed and cells were incubated with 1 μM of LysoTracker, MitoTracker, and ER-Tracker, and/or 0.1 mg/mL dextran for 30 min. The acidity of the vacuolar compartments induced by PS-NPs was confirmed using LysoSensor^TM^ Yellow/Blue DND-160. The cells were washed twice to remove the tracer, the medium was replaced with FlouroBrite^TM^ live cell fluorescence imaging medium (Gibco), and the cells were observed using a confocal microscope (ZEISS LSM 800) or captured using Cytation 5 (Biotek, Agilent Technology). The cells were stained with Hoechst 33258 for nuclear staining.

### Immunoblot analysis and immunostaining

Cultured cells were washed with PBS, fixed with 4% paraformaldehyde for 30 min at 25 ºC, permeabilized with 0.25% Triton X-100, and stained with various antibodies. Immunoblotting was performed as described previously 31. Protein expression was quantified relative to that of the endogenous loading control β-actin. The immunoreactive bands were analyzed using a chemiluminescence imaging system (UVITEC, UK; MINI H9) and ImageJ software (National Institutes of Health). Immunofluorescent images were captured using a confocal microscope (Zeiss, Germany; LSM 800).

### Statistical analysis

Statistical significance was assessed using Tukey's multiple comparison tests or Student's t-tests following one-way analysis of variance using GraphPad Prism 9 software (San Diego, CA, USA). The error bars represent the standard error of the mean. Asterisks denote *p* values less than 0.05, 0.01, 0.001, or 0.0001.

## Results

### Oral administration of PS-NPs severely aggravates steatohepatitis only in HFD-fed mice

**Figure 1:**
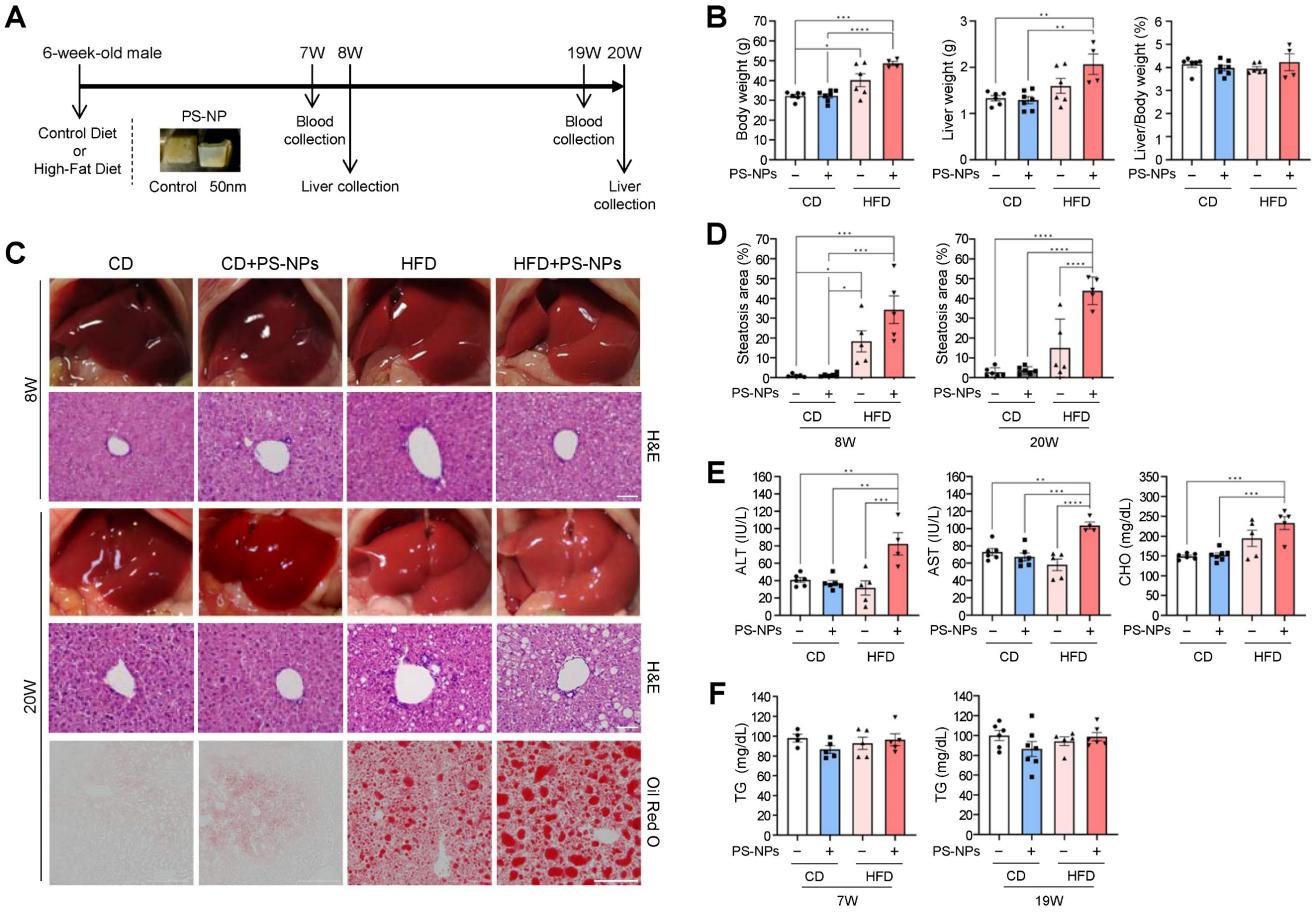
***In vivo* effects of carboxyl-modified polystyrene nanoplastics (PS-NPs) on the development of metabolic dysfunction-associated steatotic liver disease (MASLD) in high-fat diet (HFD)-fed mice. (A)** Experimental design: Mice were randomly divided into four groups and fed either a control diet with a control jelly cube (CD, n=6), a control diet with 0.5 mg of PS-NPs in a jelly cube (CD+PS-NPs, n=6), a high-fat diet containing 45 kcal% fat with a control jelly cube (HFD, n=6), or HFD with a PS-NP-jelly cube (HFD+PS-NPs, n=6) daily for 8 or 20 weeks. **(B)** Body weight, liver weight, and liver-to-body ratio in mice after 20 weeks of PS-NPs and/or HFD treatment. **(C)** Representative photographs showing liver colors in mice after 8 or 20 weeks of treatment. Hepatic lipid accumulation was assessed through hematoxylin and eosin (H&E) and Oil Red O staining. Scale bar: H&E = 50 μm, Oil Red O = 200 μm. **(D)** Quantification of Oil Red O-positive areas using ImageJ software. **(E, F)** Alanine aminotransferase (ALT), aspartate aminotransferase (AST), cholesterol (CHO), and triglyceride (TG) levels in the serum of mice after 19 weeks of treatment. Error bars represent the standard error of the mean (SEM). Asterisks indicate the *p* values: * *p* < 0.05, ** *p* < 0.01, *** *p* < 0.001, and **** *p* < 0.0001 by one-way analysis of variance (ANOVA) and Tukey's multiple comparison tests. Comparisons are with the CD group unless otherwise noted.

To assess the influence of orally ingested PS-NPs on the development of MASLD, we compared their short- and long-term effects in mice fed normal CD and those fed HFD. Mice were randomly assigned to four groups, namely CD, CD+PS-NPs, HFD, and HFD+PS-NPs; each group underwent dietary interventions for 8 or 20 weeks (Fig. 1A). Similar to what was observed in previous studies 13, mice fed a HFD for 20 weeks exhibited increased body and liver weights, indicative of hepatic steatosis. In particular, the HFD group demonstrated a 25% increase in body weight and a 20% increase in liver weight compared with the CD group, whereas the HFD+PS-NPs group displayed a 37% increase in body weight and a substantial 61% increase in liver weight compared with the CD+PS-NPs group. This effect was particularly significant in mice exposed to HFD and PS-NPs concurrently (Fig. 1B). However, in the 8-week experiment, only body weight significantly increased (Fig. S1A). These findings suggested that PS-NPs promoted weight gain and exacerbated hepatic steatosis in mice on an HFD. To further understand the impact on liver tissue, the extent of fatty changes was assessed by staining three parts of liver tissue, including the left lateral and medial lobes, with hematoxylin and eosin (H&E) or Oil Red O solution. The liver tissues from CD and CD+PS-NPs mice displayed no dramatic changes in fat accumulation or other steatosis-related pathological lesions, while HFD and HFD+PS-NPs mice showed steatosis-like aspects (Fig.1C, 1D, and S1B). In addition, oral administration of PS-NPs induced hepatic inflammation in CD-fed mice as well as HFD-fed mice, as evidenced by increased macrophages (Fig. S1C and S1D) and neutrophils (Fig. S1E and S1F), markers of chronic inflammation. Since the mouse model used in this study was chronic liver disease model, infiltration of hepatic neutrophils, an acute inflammation marker, was slightly increased in CD+PS-NPs and HFD+PS-NPs fed mice compared to CD or HFD only group (Fig. S1E and S1F). Based on this observation, we speculated that HFD exerts a synergistic effect with PS-NPs-induced inflammation to promote hepatic steatosis. Furthermore, 20 weeks of HFD exhibited zonal aggregates of mainly microvesicular hepatocytes, whereas HFD+PS-NPs group had diffuse lesions containing a mixture of macrovesicular and microvesicular hepatocytes (Fig. S1B). These findings indicate that the combination of HFD and PS-NPs led to more advanced hepatic steatosis than HFD alone in both 8- and 20-week animal experiments. Moreover, the mice fed both HFD and PS-NPs exhibited significantly increased serum ALT, AST, and CHO levels than those in CD, CD+PS-NPs, and HFD alone (Fig. 1E), but no notable differences were observed in serum triglyceride (TG) levels between each group (Fig. 1F). In contrast, 8-week of oral PS-NPs administration was not enough to affect the levels of ALT, AST, and CHO (Fig. S1G). Subsequently, we also performed Picro-Sirius Red staining analysis to visualize liver fibrosis. The exposure to PS-NPs showed increased collagen deposition in CD or HFD fed mice compared to those in the CD-alone and HFD-alone groups (Fig. S1H and S1I). Taken together, we concluded that the combination of PS-NPs and HFD aggravates hepatic steatosis.

### PS-NPs induces substantial changes in actin organization, cell-cell adhesion, and lipid metabolism

**Figure 2:**
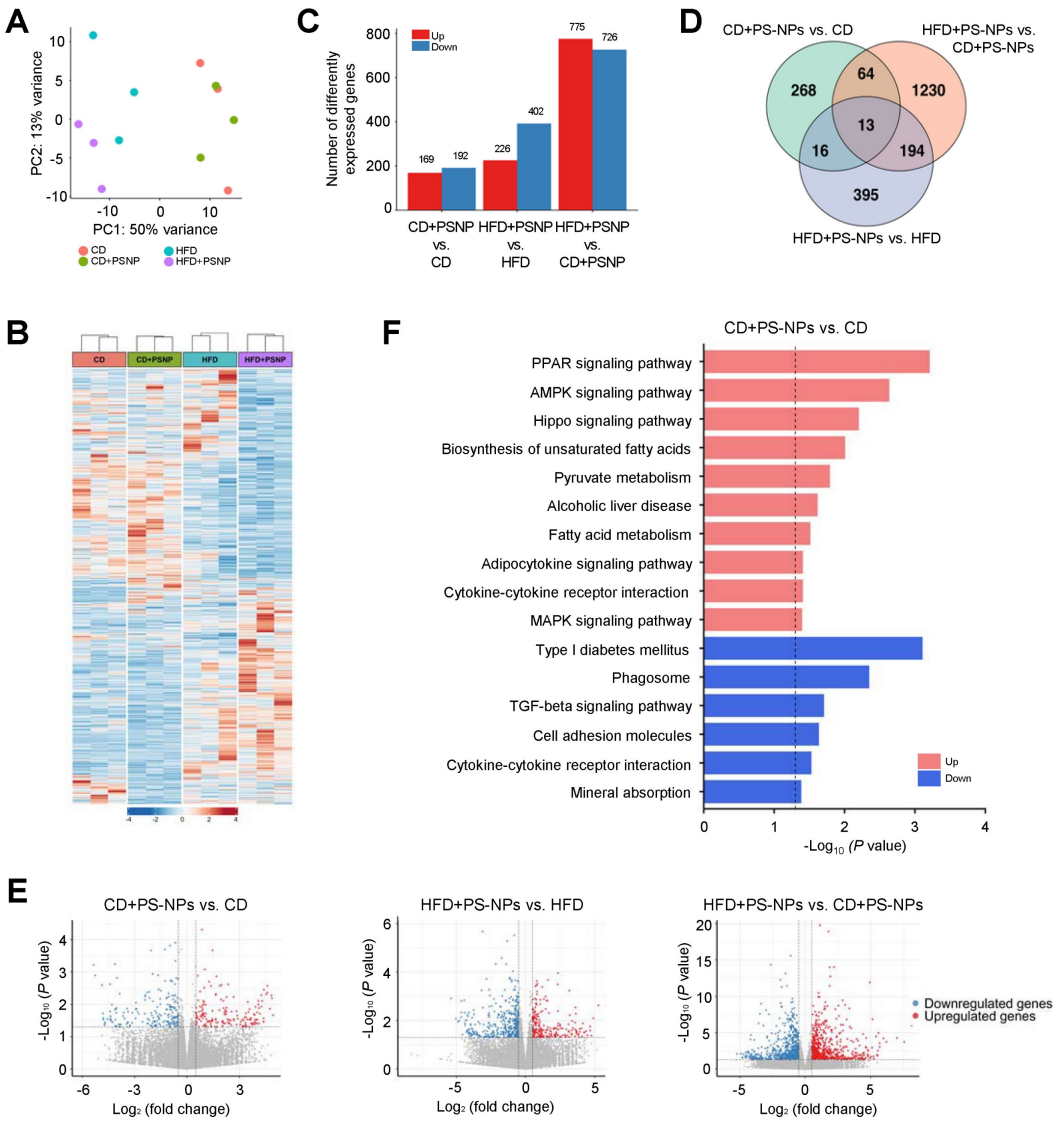
** Effect of PS-NPs on the transcriptome of HFD-fed mice over 20 weeks. (A)** Principal component analysis (PCA) plot showing gene expression data obtained by RNA sequencing analysis for the four mouse groups. **(B)** Heatmap displaying normalized gene expression for 2,180 DEGs. Each column represents a sample, and each row represents an individual gene. The color scale is based on a z-score value from -4 (blue) to 4 (red). **(C)** Number of upregulated (red) and downregulated (blue) differentially expressed genes (DEGs) in CD+PS-NPs vs. CD, HFD+PS-NPs vs. HFD, and HFD+PS-NPs vs. CD+PS-NPs (*p* value < 0.05 & |log2 FC| > 0.5). **(D)** Venn diagrams illustrating the common and unique DEGs among the three comparative pairs. **(E)** Volcano plots of DEGs in the three comparative pairs. Pink dots and blue dots present upregulated and downregulated gene expression, respectively. (F) Enriched KEGG pathways for DEGs of CD+PS-NPs vs. CD. The dotted line represents the *p* value threshold of 0.05. The pink bar presents upregulated pathways, and the blue bar presents downregulated pathways.

Differentially expressed genes (DEGs) associated with MASLD induced by PS-NPs and/or HFD were identified by hepatic transcriptome analysis of the four groups. Principal component analysis and heat map analysis revealed clear group clustering, particularly in the control and HFD-fed groups (Fig. 2A and 2B). DEGs were selected under the following conditions:* P* value < 0.05, |log2 fold change| > 0.5 (Fig. 2 and S2), resulting in 361 DEGs in the CD+PS-NPs versus the CD group; 628 DEGs in the HFD+PS-NPs versus the HFD group; and 1501 DEGs in the HFD+PS-NPs versus CD+PS-NPs group (Fig. 2C). Compared to the CD group, the CD+PS-NPs group exhibited 169 upregulated and 192 downregulated genes. Similarly, relative to the HFD group, the HFD+PS-NPs group presented 226 and 402 genes with upregulated and downregulated expression, respectively. Notably, compared with the CD+PS-NPs group, the HFD+PS-NPs group displayed 775 and 726 genes with upregulated and downregulated expression, respectively (Fig. 2C). A union set of 2180 DEGs was identified across the three comparative pairs (CD+PS-NPs versus CD, HFD+PS-NPs versus HFD, and HFD+PS-NPs versus CD+PS-NPs) in as illustrated in the Venn diagram (Fig. 2D). The two comparative pairs (CD+PS-NPs versus CD and HFD+PS-NPs versus HFD) shared 16 DEGs, including 11 coding proteins related to immune regulation and actin organization, such as *Ctse*, *Il7r*, *Cd46*, *Mt1*, *Ophn1*, and *Myom3* (Fig. 2D and S2). Consistent with our inflammation-related experiments, we found that genes involved in inflammatory responses were significantly enriched following PS-NPs treatment (Fig. S2C and S2D). However, fibrosis-related genes were not significantly altered (Fig. S2E), suggesting that the concentration and duration of HFD exposure applied in this study were insufficient to fully induce liver fibrosis. Furthermore, the DEGs identified in the three comparative pairs were associated with various processes, including the PPAR signaling pathway, lipid metabolism (biosynthetic and catabolic processes), cell-cell adhesion, and actin organization (Fig. 2E, 2F, and S2). In our study, we observed alterations in lipid metabolism and the PPAR signaling pathway. Additionally, changes in autophagy regulation, cell-cell adhesion, and cytoskeleton regulation were noted.

### PS-NPs regulates expression of lysosomal proteins in HepaRG cells

**Figure 3:**
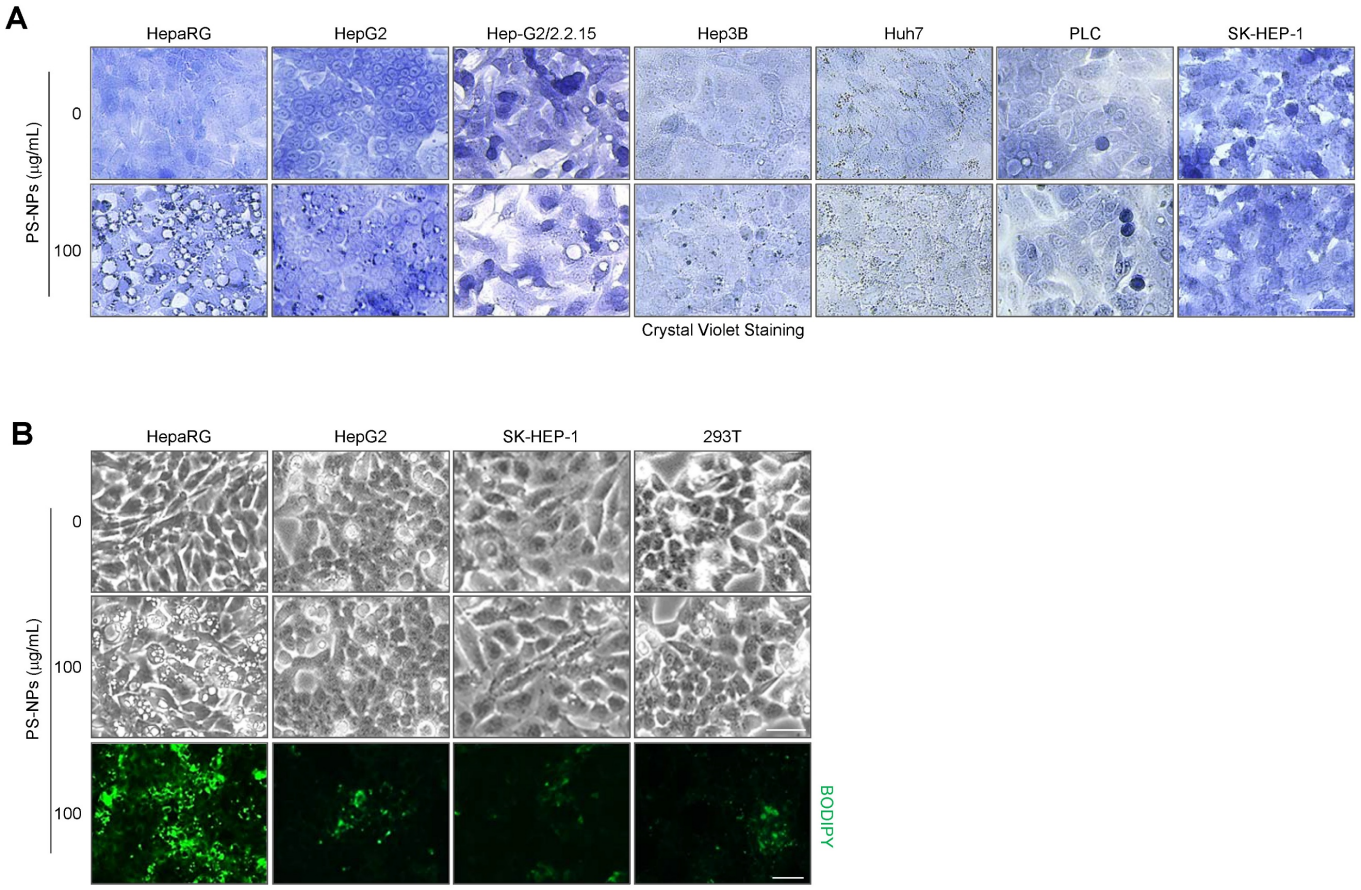
** Vacuole formation and lipid accumulation in PS-NP-treated liver cell lines. (A)** Crystal violet staining was performed to confirm vacuole formation in response to 50 nm PS-NPs in indicated hepatocyte cell lines. Scale bar: 50 μm. **(B)** BODIPY staining in four cell lines (HepaRG, HepG2, SK-HEP-1, and 293T) treated with 100 μg/mL PS-NPs, demonstrating prominent lipid accumulation in HepaRG cells. Images were captured by a Leica microscope. Scale bar: 50 μm.

We sought to corroborate these *in vivo* findings and explored the underlying mechanisms through a series of *in vitro* experiments. To investigate the detailed mechanisms of hepatic steatosis induced by PS-NP exposure, we used various hepatocyte cell lines, including HepaRG, HepG2, and SK-Hep1, as well as the 293T kidney cell line, and subsequently, analyzed cytoplasmic vacuolization and lipid accumulation. The formation of large vacuoles and a substantial increase in lipid accumulation were only observed in HepaRG cells (Fig. 3); therefore, we performed further analyses using HepaRG cell line.

**Figure 4:**
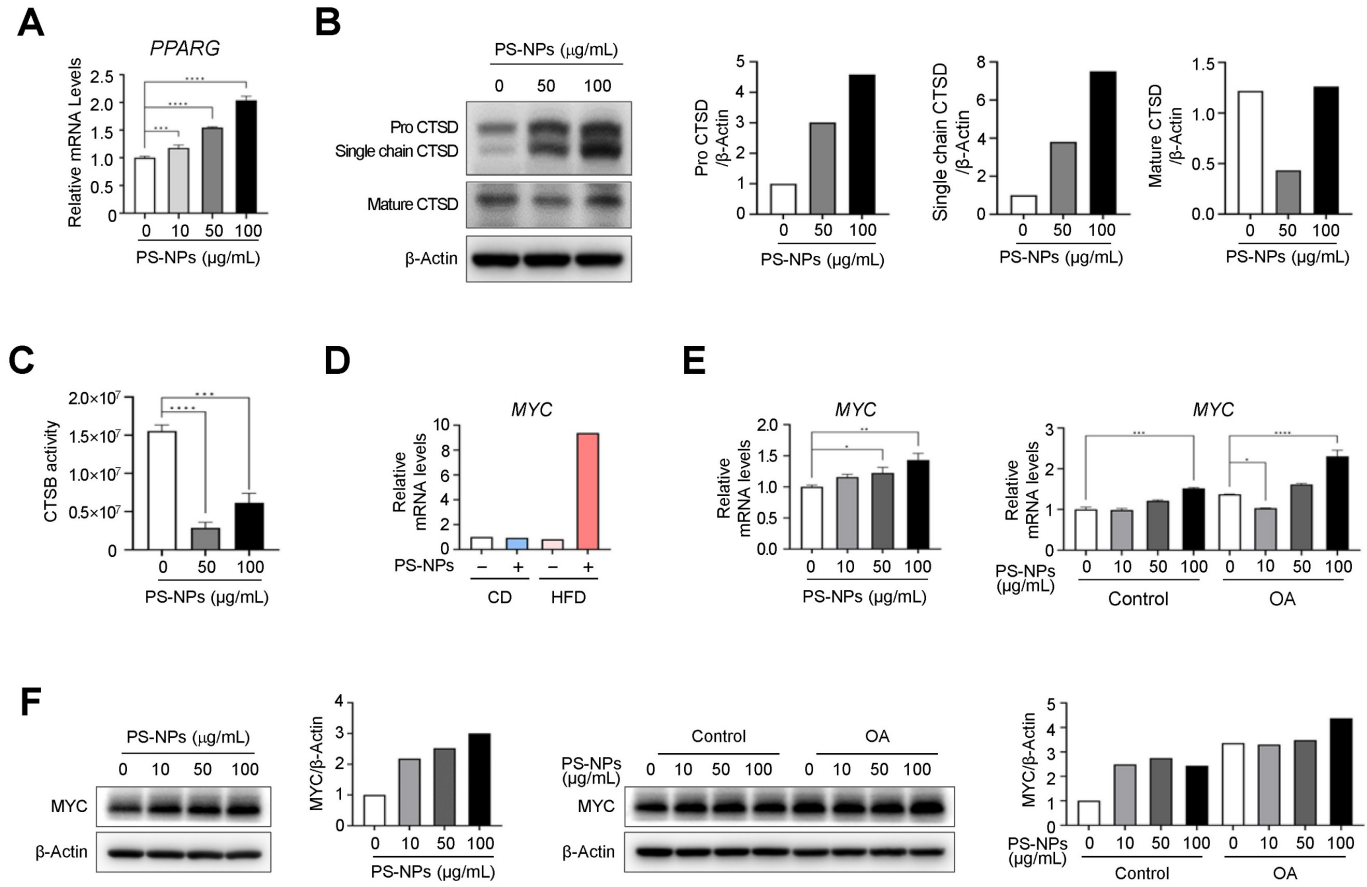
** PS-NPs treatment upregulate MYC expression levels but downregulate cathepsin activities in HepaRG cells. (A)** mRNA levels of PPARG in HepaRG cells with indicated concentration of PS-NPs. **(B)** Western blot analysis of mature cathepsin D (CTSD, active protease) and immature pro-CTSD and single-chain CTSD, showing attenuation of CTSD maturation by PS-NPs. **(C)** Attenuation of the enzymatic activity of cathepsin B (CTSB). **(D)** Relative mRNA expression level of MYC in liver tissues from 20 weeks of CD, CD+PS-NPs, HFD, and HFD+PS-NPs fed mice. **(E)** Relative mRNA expression level of MYC and (F) protein levels in HepaRG cells after exposed to various concentration of PS-NPs (left panel) or with or without 0.2mM OA (right panel) for 24 h. Error bars represent the SEM. * *p* < 0.05, ** *p* < 0.01, *** *p* < 0.001 by one-way ANOVA and Tukey's multiple comparison tests.

Our DEG analysis in Figure 2 showed PS-NPs are correlated with PPAR gamma signal pathway, MYC, and lysosomal function-related genes, such as cathepsin E (CtsE), and genes related to lipogenesis. To elucidate whether these increases are also observed *in vitro*, we first measured the expression levels of PPARG and cathepsins in PS-NPs treated HepaRG cells. As expected, PPARG mRNA levels were significantly increased in dose-dependent manner with PS-NPs (Fig. 4A). Subsequently, the levels of cathepsin D (CtsD) and B (CtsB), typical lysosomal proteins which are abundant in the liver, were analyzed to elucidate the mechanism of PS-NP-induced lysosomal dysfunction. The protein levels of CtsD were proportionally increased, but predominantly existed in immature forms, pro-CtsD and single chain CtsD, along with a concurrent decrease in the levels of mature form of CtsD (Fig. 4B). Additionally, the activity of the lysosomal enzyme CtsB was also reduced by more than 80% (Fig. 4C). Hence, our results suggested that impaired lysosomal function, marked by reduced cathepsin activity, played an important role in the onset and progression of liver disease. Furthermore, DEG data also indicated that MYC, which is highly expressed in chronic liver diseases like MASLD and hepatocellular carcinoma (HCC) 32-34, was significantly increased in mouse liver tissue obtained from HFD+PS-NPs group (Fig. S2 and 4D). Consistently, the expression levels of MYC in HepaRG cells were also increased in concentration-dependent manner with PS-NPs, and this increase was further pronounced following OA treatment (Fig. 4E and 4F). These results suggested that MYC could be another key factor in lysosomal dysfunction and MASLD.

### PS-NPs increase acidic vesicles and stimulate lipid accumulation in HepaRG cells

**Figure 5:**
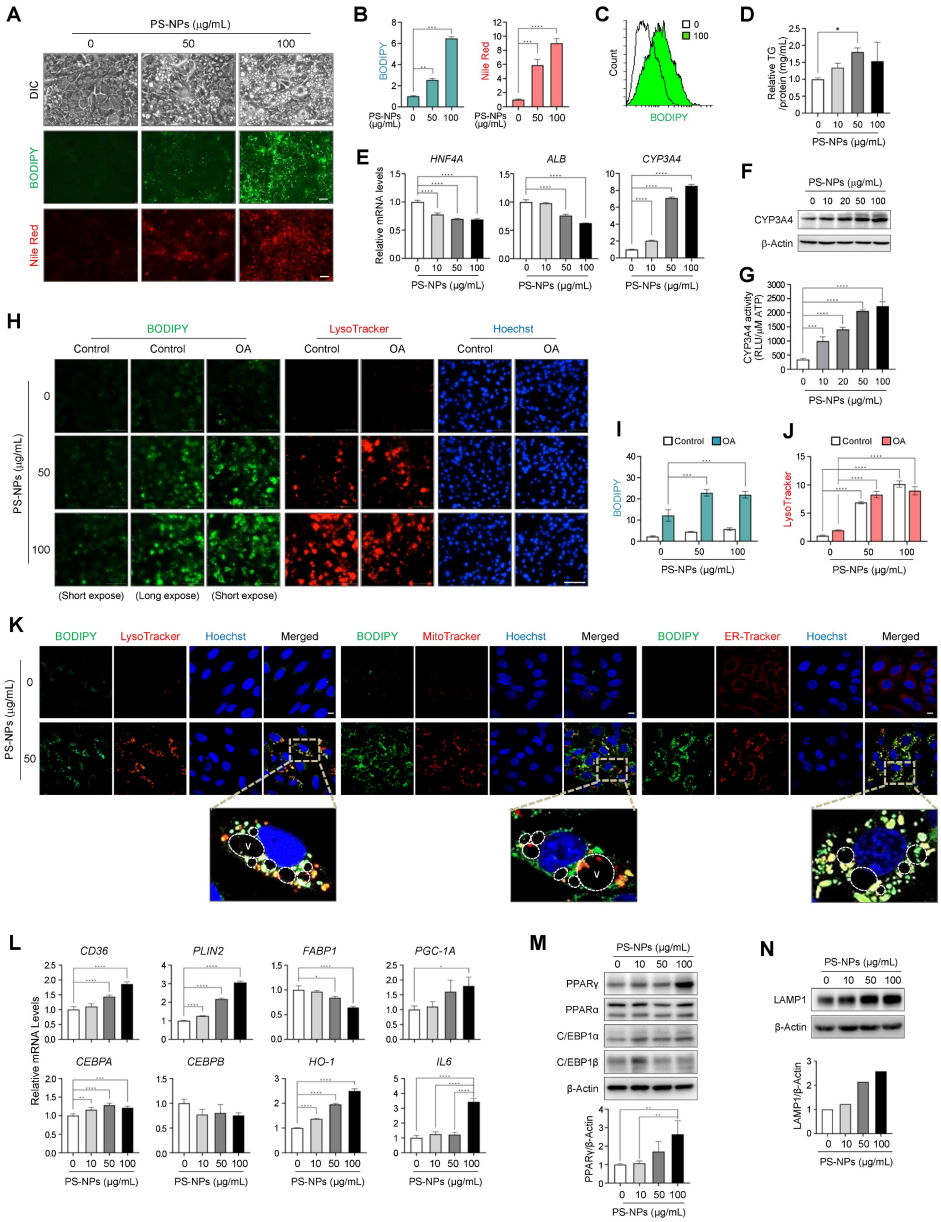
** Increased acidic compartments, lipid accumulation, and modulation of lipogenesis and lipolysis in PS-NP-treated HepaRG cells. (A)** BODIPY and Nile Red staining were used to visualize neutral lipid accumulation in HepaRG cells treated with different PS-NP concentrations for 48 h. Scale bar: 50 μm. **(B)** Quantification of cellular fluorescence intensity for BODIPY and Nile Red staining. **(C)** Histogram overlay of flow cytometry BODIPY fluorescence measurements. **(D)** Intracellular triglyceride (TG) levels in HepaRG cells after 24 h of PS-NP exposure. Error bars represent the SEM. * *p* < 0.05 by ANOVA two-tailed unpaired t-test. **(E)** mRNA expression of hepatocyte nuclear factor 4 alpha (*HNF4A*), albumin (*ALB*), and cytochrome P450 3A4 (*CYP3A4*) after 48 h of PS-NP incubation, normalized to *RPL13A* expression relative to 0 μg/mL PS-NPs. **(F)** Representative western blot images of CYP3A4 in HepaRG cells. **(G)** CYP3A4 activities in HepaRG cells exposed to varying PS-NP concentrations for 48 h. **(H)** Influence of 3-h pretreatment with 0.2 mM oleate on PS-NP-induced lipid accumulation. Scale bar: 200 μm. **(I-J)** Bar graphs showing the cellular fluorescence intensity of BODIPY (I) and LysoTracker (J) quantified using Gen5 software. Comparisons are made with the 0 μg/mL PS-NPs in with or without 0.2 mM oleate group. **(K)** Colocalization analysis of BODIPY with various organelle-specific markers (LysoTracker, MitoTracker, or ER-Tracker) in PS-NP-treated HepaRG cells. Images were acquired using a confocal microscope. Scale bar: 10 μm. **(L-N)** mRNA and protein levels of various genes associated with MASLD development. Error bars represent the SEM. * *p* < 0.05, ** *p* < 0.01, *** *p* < 0.001, **** *p* < 0.0001 by one-way ANOVA and Tukey's multiple comparison tests. **(N)** Dose-dependent increase in lysosomal membraned protein LAMP1 level following PS-NP exposure.

A previous study also used HepaRG cells as a cell-based model of lipid-induced hepatic steatosis, wherein they were treated with palmitate alone or a combination of palmitate and oleate 32. Thus, all subsequent *in vitro* experiments were performed using HepaRG cells. A dose-response experiment was conducted to confirm the appropriate concentration of PS-NPs, revealing that the IC_50_ value exceeded 1,000 μg/mL (Fig. S3). HepaRG cells exhibited concentration-dependent lipid accumulation upon exposure to PS-NPs, as illustrated in Figure 5A-5C. Additionally, TG levels were modestly increased in HepaRG cells exposed to PS-NPs (Fig. 5D).

As anticipated, the expression of *HNF4A* and *ALB* decreased, while *CYP3A4* mRNA and protein levels, as well as CYP3A4 activity increased in a dose-dependent manner upon PS-NP exposure (Fig. 5E-5G). Concurrent treatment with PS-NPs and oleate resulted in greater lipid accumulation compared to treatment with PS-NPs alone, consistent with the observations from our *in vivo* studies (Fig. 5H and 5I). Notably, lipid accumulation and lysosome numbers increased concurrently with PS-NP treatment. However, oleate was not associated with the PS-NP-induced lysosomal changes (Fig. 5H-5J). To analyze the association between nascent vacuoles and lipid droplets, HepaRG cells exposed to PS-NPs were co-stained with organelle markers. Given that neutral lipid droplets are synthesized in the endoplasmic reticulum (ER) 35, most lipids generated by PS-NPs colocalized with ER-Tracker, with some lipid droplets located in acidic organelles and others within the mitochondria (Fig. 5K). Notably, the large vacuoles induced by PS-NPs engulfed lipid droplets or fused with acidic vacuoles (Fig. 5K).

To explore the signaling mechanism underlying PS-NP-induced MASLD, we examined the expressions of several proteins associated with lipid metabolism in PS-NP-treated cells. As shown in Fig. 5L-M, we observed upregulation of genes involved in lipogenesis, including peroxisome proliferator-activated receptor gamma (*PPARG*). Additionally, *CD36*, perilipin 2 (*PLIN2*), and PPARG coactivator 1α (*PGC-1A*) and upregulated in PS-NP-treated cells. The mRNA and protein expression of CCAAT/enhancer binding protein α (*C/EBPA*), a target gene of PPARγ, was also increased. Conversely, the expression of fatty acid-binding protein 1 (*FABP1*), which was inhibited by CEBPA and associated with MASLD, was decreased 36. In addition, the expression of CCAAT/ enhancer binding protein β (*C/EBPB*), a gene related to liver regrowth and regeneration, was not significantly changed. In addition, the expression of two key oxidative regulators, heme oxygenase 1 (*HO-1*) and *IL-6*, were increased in the PS-NP-treated HepaRG cells (Fig. 5L). Consistent with increased LysoTracker staining, LAMP1 protein expression also increased in a PS-NP concentration-dependent manner (Fig. 5N). Together, these findings suggest that PS-NP treatment influences the PPARγ pathway and oxidative stress response, leading to lipid accumulation in HepaRG cells.

### Large vacuoles induced by PS-NPs contain Rab7, Lamp1 and LGP85 due to the fusion of lysosomal compartments with endocytic vesicles facilitated by actin rearrangement

**Figure 6:**
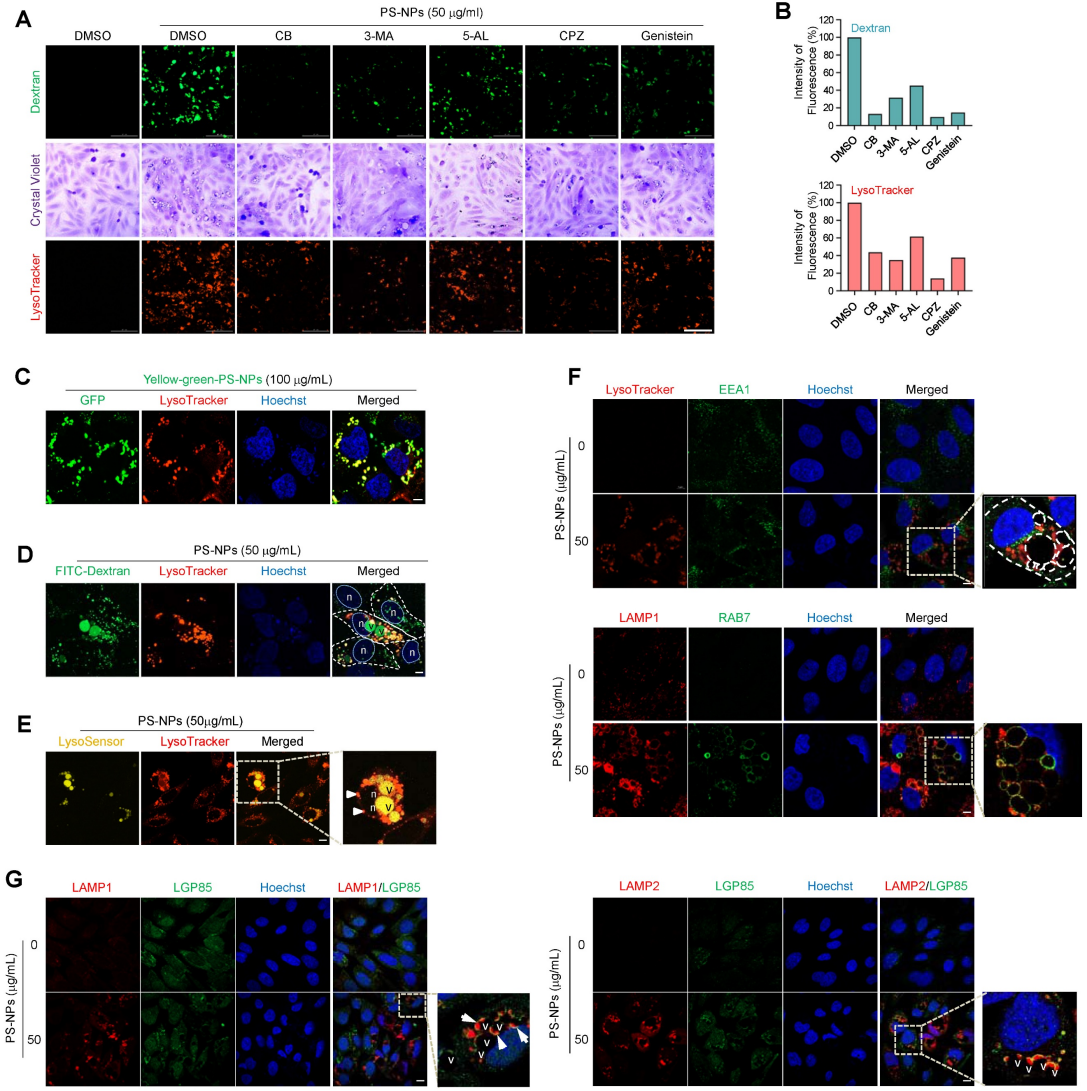
** Cellular uptake mechanism, endocytic membrane trafficking, and lysosomal accumulation of PS-NPs. (A)** HepaRG cells were pre-treated with 5 mM 3-methyladenine (3-MA), a PI3K inhibitor; 1 μM cytochalasin B (CB), an inhibitor of actin polymerization; 5 μM 5-(N-ethyl-N-isopropyl) amiloride (5-AL), a micropinocytosis inhibitor; 5 μM chlorpromazine hydrochloride (CPZ), a clathrin-mediated endocytosis inhibitor; or 20 μM genistein, a caveolae-mediated endocytosis inhibitor, followed by treatment with 0 or 50 μg/mL PS-NPs for 24 h. Next, cells were stained with 0.5% crystal violet for morphological observation or incubated with 0.1 mg/mL dextran and 1 μM LysoTracker for 30 min. The images were captured using Cytation5. Scale bar: 200 μm. **(B)** Bar graphs display the cellular fluorescence intensity of dextran or LysoTracker quantified using Gen5, and each intensity is normalized to the cell count. **(C)** Visualization of PS-NP accumulation in the acidic compartment using GFP-conjugated PS-NPs (PS-NP-GFP). Scale bar: 5 μm. **(D)** Internalization analysis of PS-NPs using fluorescein-conjugated dextran. PS-NP-treated HepaRG cells were incubated with dextran (green) and LysoTracker (red) for 30 min. Scale bar: 5 μm. n; nucleus, v; vacuoles **(E)** pH assessment of large vacuoles. PS-NP-exposed HepaRG cells were stained with 1 μM LysoSensor^TM^ Yellow/Blue DND-160 (yellow fluorescence in acidic organelles) and with LysoTracker to confirm vacuole acidity. Nuclei were stained with Hoechst 33258. Scale bar: 10 μm. n; nucleus, v; vacuoles. **(F)** Identification of large vacuolar compartments generated by PS-NP exposure using organelle markers: LysoTracker (acidic organelles), EEA1 (early endosomes), LAMP1 (lysosomes), and RAB7 (late endosomes). Scale bar: 5 μm. **(G)** Upregulation of lysosomal membrane proteins, LAMP1, LAMP2, and LGP85, and their colocalization on vacuole membranes induced by PS-NPs. Scale bar: 10 μm.

To elucidate the mechanisms and trafficking processes of vacuoles generated by PS-NPs, endocytic uptake was observed using an endocytosis inhibitor and dextran. Initially, various inhibitors were used to modulate cellular uptake processes (Fig. 6A). Each inhibitor reduced the uptake of 50 nm PS-NPs by more than 50%. Inhibition of actin-, caveolin-, and clathrin-mediated endocytosis significantly decreased the internalization of 50 nm PS-NPs by more than 80%. Regarding the impact of inhibitors on the acidic organelles induced by PS-NPs, CPZ, an inhibitor of clathrin-mediated endocytosis, exhibited the most pronounced effect, with more than 80% inhibition in HepaRG cells (Fig. 6A and 6B). Therefore, our findings suggest that the primary pathways for internalization of PS-NPs involve clathrin-mediated endocytosis by actin rearrangement 37, with macropinocytosis playing a relatively minor role (Fig. 6A and 6B). Experiments using Yellow Green-conjugated PS-NPs revealed that PS-NPs taken up via endocytosis primarily colocalized with LysoTracker, a marker for acidic compartments containing endosomes, lysosomes, and autophagic vacuoles (Fig. 6C).

As noted above, PS-NP-treated cells formed large vacuoles that frequently engulfed lipid droplets or fused with acidic vesicles, often attaching to the edges of the large vacuoles (Fig. 5K). Investigation of intracellular trafficking using the fluorescent tracer FITC-dextran established that the large vesicles originated from the fusion of endocytosed vesicles (Fig. 6D and S4). Additionally, a live imaging assay using LysoSensor DND-160, which produces yellow fluorescence in an acidic environment 38, demonstrated the acidic pH of these large vacuoles, as illustrated in Figure 6E. To understand the process of large vacuole formation, the colocalization of various membrane proteins within large vacuoles was investigated during endocytosis. EEA1, a marker for the early endosomes 39 did not co-localize with large vacuoles. In contrast, RAB7, a marker of the late endosome 40, and LAMP1, a lysosomal marker, colocalized with the membranes of large vacuoles, indicating the fusion of endo-lysosomal vesicles between the late endosomes and lysosomes (Fig. 6F) 40, 41. LGP85 is a major lysosomal membrane protein in the mouse liver 42 and is believed to play a role in the biogenesis of endolysosomes (ELs) through processes such as fusion and fission 43. We observed the expression of LGP85 (shown in green), which was overexpressed in enlarged endosomal/lysosomal compartments, was upregulated and colocalized with LAMP1 and LAMP2 expression (displayed in red) at the membrane of PS-NPs-mediated vacuoles in HepaRG cells exposed (Fig. 6G). Therefore, we concluded that the accumulation of PS-NPs may disrupt lysosomal function, increasing the expression of lysosomal proteins and the formation of enlarged vacuoles (e.g., ELs and autolysosomes [ALs]).

### PS-NPs accumulate within lysosomes, impeding autophagic flux, and incomplete autophagy leads to the formation of large vacuoles and subsequent fat accumulation

**Figure 7:**
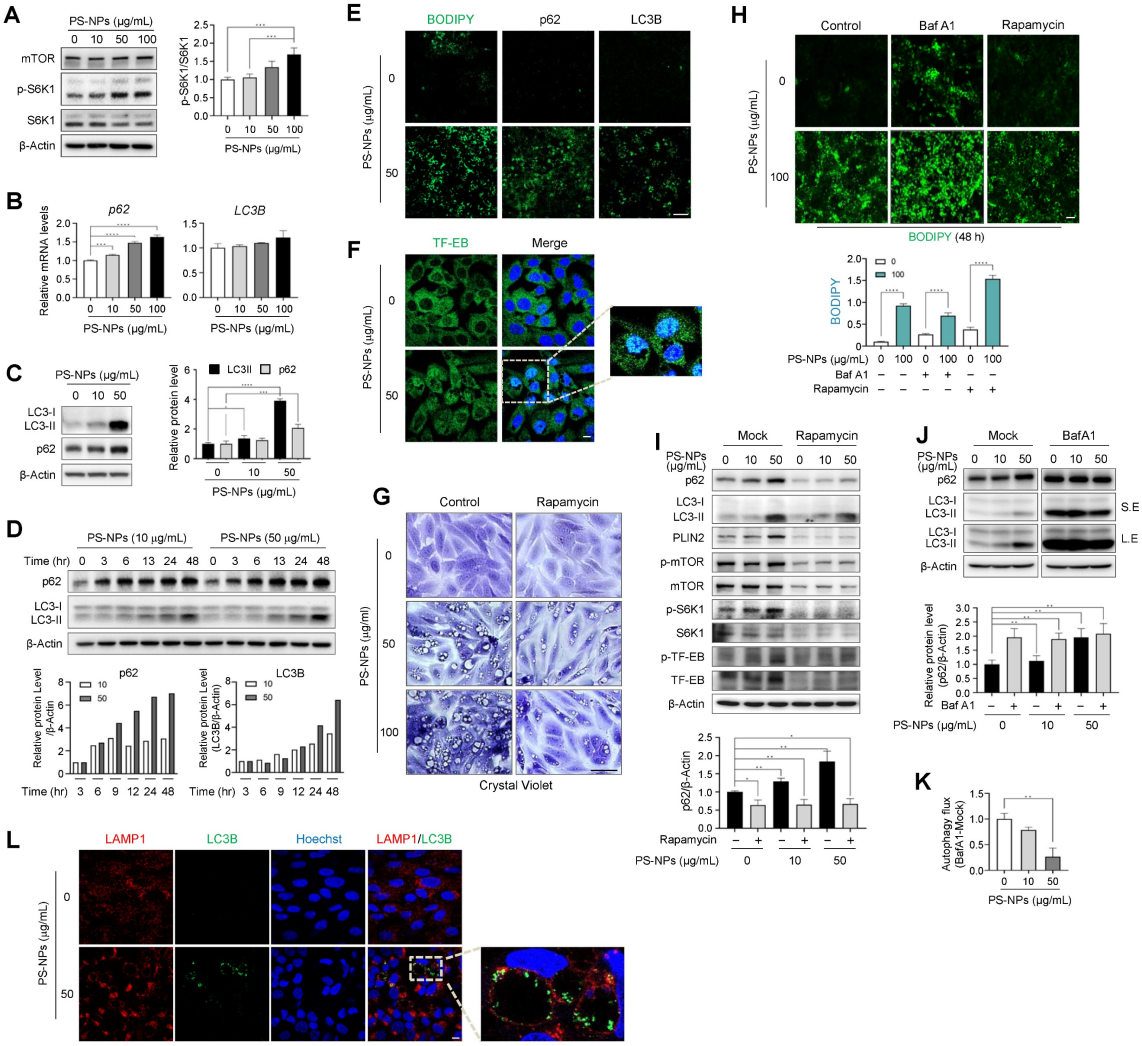
** Disturbance of autophagy flux induced by PS-NPs in MASLD development. (A)** Increased S6 kinase 1 (S6K1) phosphorylation, an mTORC1 substrate. **(B, C)** mRNA (B) and protein (C) expression of p62, an autophagy substrate, and LC3B, a marker of autophagosomes. **(D)** Time-dependent protein expression of p62 and LC3B in PS-NP-treated HepaRG cells. Bar graphs show the intensity of each protein quantified using ImageJ software and normalized to β-actin; data are presented as the fold change relative to the 0 h sample. **(E)** Enhanced BODIPY-stained lipid accumulation induced by PS-NPs, accompanied by elevated immunofluorescence of p62 and LC3B. Scale bar: 50 μm. **(F)** Translocation to the nucleus of transcription factor EB (TFEB), an mTORC1 substrate. Scale bar: 10 μm. **(G)** Inhibition of PS-NP-induced vacuolization by rapamycin. HepaRG cells were pre-treated with 2 μg/mL rapamycin, followed by PS-NP exposure for 24 h, and then stained with crystal violet. Images were captured by a Leica microscope. Scale bar: 50 μm. **(H)** Changes in lipid accumulation (BODIPY) following treatment with autophagy regulators. HepaRG cells were pre-treated with 2 μg/mL rapamycin (an autophagy activator), followed by 10 nM Bafilomycin A1 (Baf A1; an autophagy inhibitor) for 3 h, and then PS-NPs. Scale bar: 50 μm. **(I-J)** Western blot and autophagy flux assessment. Effect of rapamycin (1 μg/mL) (I) and BafA1 (10 nM) (J) on PS-NP-triggered autophagy perturbations. Bar graphs depict the intensity of the p62 protein normalized to that of β-actin. **(K)** Examination of PS-NP-induced autophagy flux disturbances. **(L)** Accumulation of LC3B (a marker of autophagosomes, green) bound to the inner membrane of LAMP1-positive endolysosomes and autophagosomes in PS-NP-exposed HepaRG cells. Scale bar: 10 μm. Error bars represent the SEM. * *p* < 0.05, ** *p* < 0.01, *** *p* < 0.001, **** *p* < 0.0001 by one-way ANOVA and Tukey's multiple comparison tests.

To investigate whether there is a correlation between the mammalian target of rapamycin complex 1 (mTORC1) and the progression of MASLD induction by PS-NPs, changes in the mTORC1 pathway and autophagy-related proteins were examined. mTORC1 is a critical signaling molecule for both lipid synthesis and removal 44 and a negative regulator of autophagy. PS-NPs induced mTOR activity by increasing the phosphorylation of the mTOR substrate S6 kinase 1 (S6K1) (Fig. 7A and S5). Furthermore, PS-NPs led to a dose- and time-dependent increase in the mRNA and/or protein levels of the autophagosome markers p62 and LC3-II (Fig. 7B-D). The elevated levels of p62 and LC3B were directly proportional to the extent of PS-NP-induced lipid accumulation (Fig. 7E). Both autophagy activation and decreased autophagic degradation cause an increase in the autophagosome markers p62 and LC3 22. Additionally, PS-NP treatment increased protein expression levels of the transcription factor EB (TFEB) in the nucleus, a critical regulator of lysosomal biogenesis 45, and translocation from the cytoplasm to the nucleus (Fig. 7F).

To confirm that the decline in autophagy was attributed to the accumulation of PS-NPs within lysosomes, cells were treated with rapamycin, an mTOR inhibitor and autophagy inducer 46, prior to exposure to PS-NPs. Rapamycin markedly reduced the occurrence of large vacuoles induced by PS-NPs (Fig. 7G), curbing the increase in phosphorylated-S6K1 and perilipin2 (PLIN2) expression (Fig. 7I and S5). As PLIN2 promotes lipid droplet formation and contributes to fat accumulation in liver, the attenuation of PLIN2 expression by rapamycin may lead to reduced lipid accumulation (Fig. 7H and 7I) 47, 48.

Furthermore, rapamycin treatment impeded the accumulation of p62 and LC3 proteins, which were otherwise heightened in response to PS-NP exposure (Fig. 7I and S5). Next, to assess the activity of autophagic flux, cells were pre-treated with bafilomycin A1 (BafA1), an inhibitor of autophagy that impedes autophagosome-lysosome fusion 23. Consequently, Baf A1 elevated lipid accumulation and p62 levels those induced by PS-NPs (Fig. 7I and 7J). In addition, the decrease in the difference in the p62 ratio based on Baf A1 treatment in the presence of PS-NPs indicate that PS-NP treatment impairs autophagic flux (Fig. 7J and 7K). These findings indicate that PS-NPs accumulate in lysosomes and disturb autophagic flux and suggest large vacuoles are a result of incomplete autophagy, accompanied by accumulation of p62 and LC3. Therefore, we assessed persistence of the LC3B in enlarged LAMP1-positive vacuoles. Expectably, LC3B was not completely degraded in cells treated with PS-NPs and accumulated at the vacuolar membrane (Fig. 7L). Typically, inner membrane-bound LC3-II undergoes degradation by lysosomal proteases upon autophagosome-lysosome fusion and lysosomal protease dysfunction most likely occurs due to the inhibition of cathepsin activity by the lipid storage materials 49. Thus, we concluded that PS-NPs can induce autophagy but disrupt its progression, as indicated by reduced autophagic flux levels and the accumulation of autophagosomes and autolysosomes.

## Discussion

The increased consumption of plastic and widespread MP/NP pollution have potential links to various human diseases due to heightened exposure, prompting increased attention to the adverse impacts of plastic products. Both inhalation and ingestion of MPs and NPs share similar systemic responses such as oxidative stress and inflammation in the liver 50, 51. Exposure to particulate matter (PM) 2.5, representative of inhaled MP size (2500 nm), exerts its effect via indirect systemic responses while NPs could be internalized directly into the cell and co-localized with lysosomes as shown in Figure 6. Interestingly, the PM2.5 exposure along with HFD did not always show discernable effects on hepatic steatosis 50, 52, suggesting the disease onset may require further stimuli or direct tissue damages. In the case of NPs, the excessive lipid from HFD may combine with NPs, forming NP-lipid corona structure 53-55. This specific structure could facilitate the cellular intake of NPs by hepatic endocytosis via the interaction between lipid-binding domains and its lipophilic phospholipid coating 54, 56, 57. As for the size of NPs, 30 to 80nm size range (50 nm for this study) could imitate ingested particulates as it is comparable size to chylomicron remnants which are the dietary form of lipids for hepatocytes 21, 57, 58**.** Dietary intake is considered as the main source of human exposure to MPs/NPs, and gastrointestinal translocation of ingested PS and engineered nanoparticles has also been demonstrated. Studies have found cellular toxicity at high concentrations of MPs/NPs 19, 55, 59-61, indicating potential adverse effects based on the cell type, particle size, and method of uptake. Ingested nanomaterials tend to accumulate in the liver and kidneys, affecting their clearance and metabolism 10, 56. Recent *in vivo* studies have shown that orally administered PS-NPs were deposited in the livers of HFD-fed mice and increased intestinal inflammation and lipid accumulation, resulting in the development of MASLD 3, 13, 57. However, some experiments involving pure MPs and NPs did not reveal significant cytotoxic effects on human cells. Therefore, the potential human health risks associated with exposure to pure MP/NPs remain controversial.

The development of chronic liver diseases is associated with the increasing prevalence of obesity worldwide. Environmental pollutants further increase the vulnerability of the liver; therefore, there has been increasing focus on the harmful effects of NPs on the liver. Our study aimed to elucidate the long-term consequences of plastic pollution on human liver health by investigating the link between NP exposure and chronic liver disease. Using a combination of *in vivo* and *in vitro* approaches, we assessed the effects of PS-COOH-NPs on steatosis and related liver diseases. First, we examined the cumulative impact of PS-NPs on experimentally induced fatty liver disease by administering daily oral doses to mice of 0.5 mg PS-NP-infused jelly cubes and a HFD for either 8 or 20 weeks. As shown in Figure 1 and S1, this combination led to a notable increase in both liver and body weights, aggravating steatohepatitis. AST and ALT levels were increased in the HFD+PS-NPs group. Elevated blood ALT levels indicated potential liver damage or disease due to the release of ALT from damaged liver cells. However, there was no marked increase in the levels of blood markers indicative of liver damage in mice that were orally administered CD+PS-NPs or fed only HFD. These results indicated that the combination of PS-NPs and HFD aggravated hepatic steatosis in mice.

In our 20-week study, KEGG analysis revealed that DEGs were involved in the PPAR signaling, AMPK signaling, and fatty acid biosynthesis pathways, consistent with previous results (Fig. 2F) 2, 3. Similar to previous study 36, we observed changes in the expression of genes associated with lipid metabolism, lipid synthesis, and lipid transport, including *CD36, PLIN2, PPARG,* and* FABP1* in *in vitro* experiments using HepaRG cells (Fig. 5L). These findings indicate that MPs/NPs can disrupt lipid metabolism, leading to lipid accumulation in aquatic organisms 1-3 and mice 4, 13. For example, in a study by Lai et al. (2021), large yellow croakers exposed to 80 nm PS-NPs presented significant accumulation of lipid droplets in the liver, and their liver TG and lipid content were considerably higher than that in the control group. Simultaneous exposure of mice to nanoparticles and HFD for 8 weeks increased gut barrier permeability 62, 63 and metabolic stress 12. Intravenous injection of NPs (42 nm, 10 or 50 μg/mouse) increased TG accumulation and fibrosis in HFD-fed mice 13. Conversely, oral exposure to NPs (500 nm at 1,000 μg/L) for 5 weeks in mice fed a regular diet reduced liver lipid levels, as previously reported 9. Although the effect of nanoparticles on hepatic lipid accumulation in a regular diet is still controversial, the simultaneous administration of an HFD and oral PS-NPs increased lipid accumulation in the liver, indicating that PS-NPs interfere with normal lipid metabolism in mice and may pose a potential risk of increased fat deposition. Opposing effects of NPs on liver lipid metabolism in HFD- and chow-fed mice may be due to variations in basal metabolic status 8, 63, 64.

The results of Gene Ontology functional annotation revealed that PS-NP exposure in mice disrupted intercellular adhesion, actin organization, immune regulation, and hepatic lipid metabolism (Fig. S2A). Notably, genes such as *Il7r*, *Ctse*, *Cd46*, *Ophn1*, *Myom3*, *Coro6*, *Tnfrsf25*, *Racgap1*, *Mecom*, *Antxr1*, and* Plekhh2* induced alterations in the liver tissues of PS-NP-treated mice compared to those of the control group (Fig. 2C). These findings suggest an association between endocytosis, cell migration, cell growth, and differentiation in response to PS-NP treatment. Furthermore, the actin remodeling plays a crucial role in endosomal movement 65, especially in endocytic uptake. Additionally, it has been reported that actin depolymerization affects NP uptake by HeLa and 1321N1 cell lines 66. In this study, we showed that endocytic pathway inhibitors reduced the uptake of 50 nm PS-NPs by more than 50%. Specifically, inhibition of caveolin- and clathrin-mediated endocytosis decreased the internalization of 50 nm PS-NPs by more than 80%. Regarding the PS-NP-induced reduction in acidic organelles, clathrin-mediated endocytosis had the most pronounced effect, with more than 80% inhibition observed in HepaRG cells (Fig. 6). Consequently, our findings in HepaRG cells indicated that clathrin-mediated endocytosis is the predominant process of internalization, and that actin organization plays a crucial role. These results suggest that the primary pathway of PS-NPs internalization involves clathrin- and caveolin-mediated endocytosis 37, with macropinocytosis playing a minor role (Fig. 6A and 6B). Importantly, PS-NPs internalization was closely associated with hepatic inflammation, as evidenced by increased expression of immune-related genes and pro-inflammatory cytokines (Fig.S2C and S2D). These results suggest that clathrin-mediated endocytosis and actin dynamics may contribute to nanoparticle-induced immune activation in the liver.

Following treatment with PS-NPs, HepaRG cells formed vesicles of various sizes that were not observed in other liver cell lines; this phenotype was accompanied by an increase in lipid accumulation and the presence of acidic organelles such as secondary lysosomes and autophagic vacuoles (Fig. 3, 5A-5C, 5H-5J, and 6E). Dextran uptake, LysoTracker, and LysoSensor staining experiments revealed that vesicles of varying sizes in HepaRG cells induced by PS-NPs contained exogenous dextran (Fig. 6D, 6E, and S4) and exhibited an acidic pH. Notably, large vesicles were marked by the absence of the early endosome marker EEA1 but were positive for the late endosome marker RAB7 and the lysosomal markers LAMP1 and LGP85 (LIMP II) (Fig. 6). Previous studies have reported that the overexpression of LGP85 resulted in significant swelling of vacuoles, displaying characteristics of both endosomes and lysosomes 43. This observation classified vesicles into ELs and ALs. Furthermore, PS-NPs and their lipid byproducts accumulated in acidic vesicles, such as ELs/ALs, increasing the number of LAMP1-positive vesicles (Fig. 5F and 5G). Our findings show that the stimulation of autophagy using the mTOR inhibitor rapamycin significantly attenuated the formation of large vesicles and the lipid accumulation triggered by PS-NPs. Moreover, substantial changes in the expression of associated proteins were observed (Fig. 7A and 7I). PS-NP treatment resulted in the accumulation of p62 and LC3B proteins (Fig. 6C and 6D).

Biomaterials within cells destined for degradation reach the lysosomes via the endocytic/phagocytic and autophagic pathways, where late endosomes fuse with lysosomes to form ELs, which are primarily responsible for degradation. The accumulation of undigested lipids can impede membrane trafficking and lysosomal enzyme delivery, which can lead to the enlargement and dysfunction of lysosomes 7. Cells adapt to lysosomal dysfunction by increasing basal autophagy and the expression of lysosomal proteins, such as LAMP1, to compensate for the reduced degradation capacity 67. Such compensatory changes may enable cells with lysosomal dysfunction to survive 68, 69. Several studies have shown defective autophagy-lysosomal function in the livers of patients with obesity and MASLD 70; in mice with hepatic steatosis, overactivation of mTOR has been observed 46, 71. Expression of the major autophagy receptor p62/SQSTM-1 increases when autophagy is disturbed, and elevated lipid loading in lysosomes correlates with altered or impaired autophagy, contributing to increased MASLD activity. LC3B is associated with autophagosomes and ALs, and the accumulation of undigested material can lead to the formation of enlarged dysfunctional lysosomes, possibly due to increased vesicular content and osmolarity 7. If lysosomal function or autophagosome-lysosome fusion is compromised, ELs, ALs, and autophagosomes may persist without degradation. This leads to the accumulation of incompletely digested material and the formation of large vesicles.

Genes encoding critical lysosomal proteins, including V-ATPase subunits and cathepsin proteases, play crucial roles in maintaining lysosomal acidity and degradation. As shown in Figure 6J-6K, exposure to PS-NPs at 50 μg/mL disrupted autophagic flux, consistent with the effects observed following treatment with the V-ATPase inhibitor, BafA1. V-ATPase-deficient lysosomes continue to fuse with autophagosomes and endosomes, leading to the formation of enlarged ALs. Furthermore, the activities of other major lysosomal proteins― the lysosomal proteases CtsB (cysteine protease) and CtsD (aspartyl protease)―were reduced in HepaRG cells (Fig. 4B and 4C). Subsequent *in vivo* RNA analysis revealed that PS-NP induced changes in the expression of CtsE, an aspartyl protease. The major lysosomal proteases, CtsB and CtsD, are widely distributed in endosomes and lysosomes and are implicated in autophagic activity 72-75. Patients with MASLD display abnormal autophagy and reduced liver expression of CtsB, CtsD, and CtsL 73. In addition, CtsE deficiency is associated with a novel form of lysosomal storage disorder in mouse macrophages 75. Saku et al. (1991) demonstrated that the expression of CtsE was localized within the bile canaliculi of the rat liver and the microvilli of hepatic cells 76.

Thus, this study suggests that impaired lysosomal function, characterized by reduced cathepsin activity and compromised autophagy, plays a pivotal role in the onset and progression of liver disease. In addition, it suggests that MYC, an oncogene, may be another therapeutic target for this phenomenon. Furthermore, our findings suggest that PS-NPs swiftly penetrate cells via the endocytic system, inducing the formation of cytosolic lipid droplets, which are subsequently delivered to lysosomes for autophagy and lipophagy. However, the nanoparticles may not undergo efficient digestion within the lysosomes, disrupting the functions of lysosomal proteins, such as V-ATPase and cathepsins, thereby impairing the degradative capabilities of these organelles and potentially compromising the critical cellular membrane turnover involved in macroautophagy. Therefore, it is plausible that mice exposed to PS-NPs experience dysfunction in autophagy and lipophagy, contributing to the development of liver steatosis and exacerbated hepatitis. Hence, experimental therapies are being explored to restore lysosomal function, focusing on cathepsin activity with the aim of ameliorating MASLD. In summary, our findings underscore the potential health risks associated with PS-NPs, particularly in accelerating obesity and fatty liver disease development under HFD conditions. Experimental therapies targeting lysosomal function, especially cathepsin activity, are under investigation to alleviate metabolic associated fatty liver disease (MASLD) 77.

However, research on the health risks posed by PS-NPs is still in the early stages, and further investigation is imperative to elucidate the mechanisms by which PS-NPs affect various cellular processes. These deficiencies highlight the need for additional research to unravel the underlying molecular pathways.

## Conclusions

Our results suggest that PS-NPs swiftly penetrated cells by inducing actin reorganization, prompting the formation of small cytosolic vesicles. These vesicles are then transported to autophagosomes and lysosomes for degradation. However, PS-NPs may hinder efficient digestion, disrupting the function of crucial lysosomal proteins such as V-ATPase and cathepsins. This impairment compromises lysosome-based degradation, potentially affecting cellular membrane turnover, micro- and macroautophagy, leading to the formation of large vacuoles within cells. Consequently, exposure to PS-NPs may disrupt lysosomal degradation, contributing to liver steatosis and exacerbating hepatitis. In conclusion, our findings underscore the health risks associated with PS-NPs, particularly in exacerbating obesity and fatty liver disease development under high-fat diet conditions.

## Supplementary Material

Supplementary figures and tables.

## Abbreviations

ALs: autolysosomes
ALT: alanine aminotransferase
AST: aspartate aminotransferase
ATP: adenosine triphosphate
BafA1: bafilomycin A1
CD: chow diet
Cd46: membrane cofactor protein
CEBPA: CCAAT enhancer binding protein α
CHO: cholesterol
CtsE: cathepsin E
DEGs: Differentially expressed genes
EEA1: early endosome antigen 1
ELs: endolysosomes
ER: endoplasmic reticulum
FABP1: fatty acid-binding protein 1
FBS: fetal bovine serum
HFD: high-fat diet
HO-1: heme oxygenase 1
IL: interleukin
KEGG: Kyoto Encyclopedia of Genes and Genomes
KRIBB: Korea Research Institute of Bioscience & Biotechnology
LAMP1: lysosomal-associated membrane protein 1
MASLD: metabolic dysfunction-associated steatotic liver disease
MPs: microplastics
Mt1: metallothionein 1
mTORC1: mammalian target of rapamycin complex 1
NPs: nanoplastics
PBS: phosphate-buffered saline
PGC-1A: PPARG coactivator 1α
PLIN2: perilipin 2
PPARG: peroxisome proliferator-activated receptor gamma
PPARγ: peroxisome proliferator-activated receptor γ
PS-NPs: polystyrene nanoplastics
PS: polystyrene
S6K1: S6 kinase 1
TFEB: transcription factor EB
TG: triglyceride
