# Supplementary material for: Polystyrene Nanoplastics Exacerbate HFD-induced MASLD by Reducing Cathepsin Activity and Triggering Large Vacuole Formation via Impaired Lysosomal Acidification
Source: Int J Biol Sci. 2025 Jun 9;21(9):3867–85. doi: 10.7150/ijbs.108268 (PMC12210384; doi:10.7150/ijbs.108268)
Supplement: Supplementary file 1 — Supplementary figures and tables. [file ijbsv21p3867s1.pdf]

SUPPLEMENTARY MATERIAL for

Polystyrene Nanoplastics Exacerbate HFD-induced MASLD by Reducing Cathepsin Activity and Triggering Large Vacuole Formation via Impaired Lysosomal Acidification

Jiwon Ahn, Kajung Ryu, Hyerin Kim, Hwi Won Seo, Minsu Jang, Seung-Hyun Kim, Yunho Park, Myung Jin Son, Ho-Joon Lee, Ok Seon Kwon, and Kyung-Sook Chung

SUPPLEMENTARY FIGURES

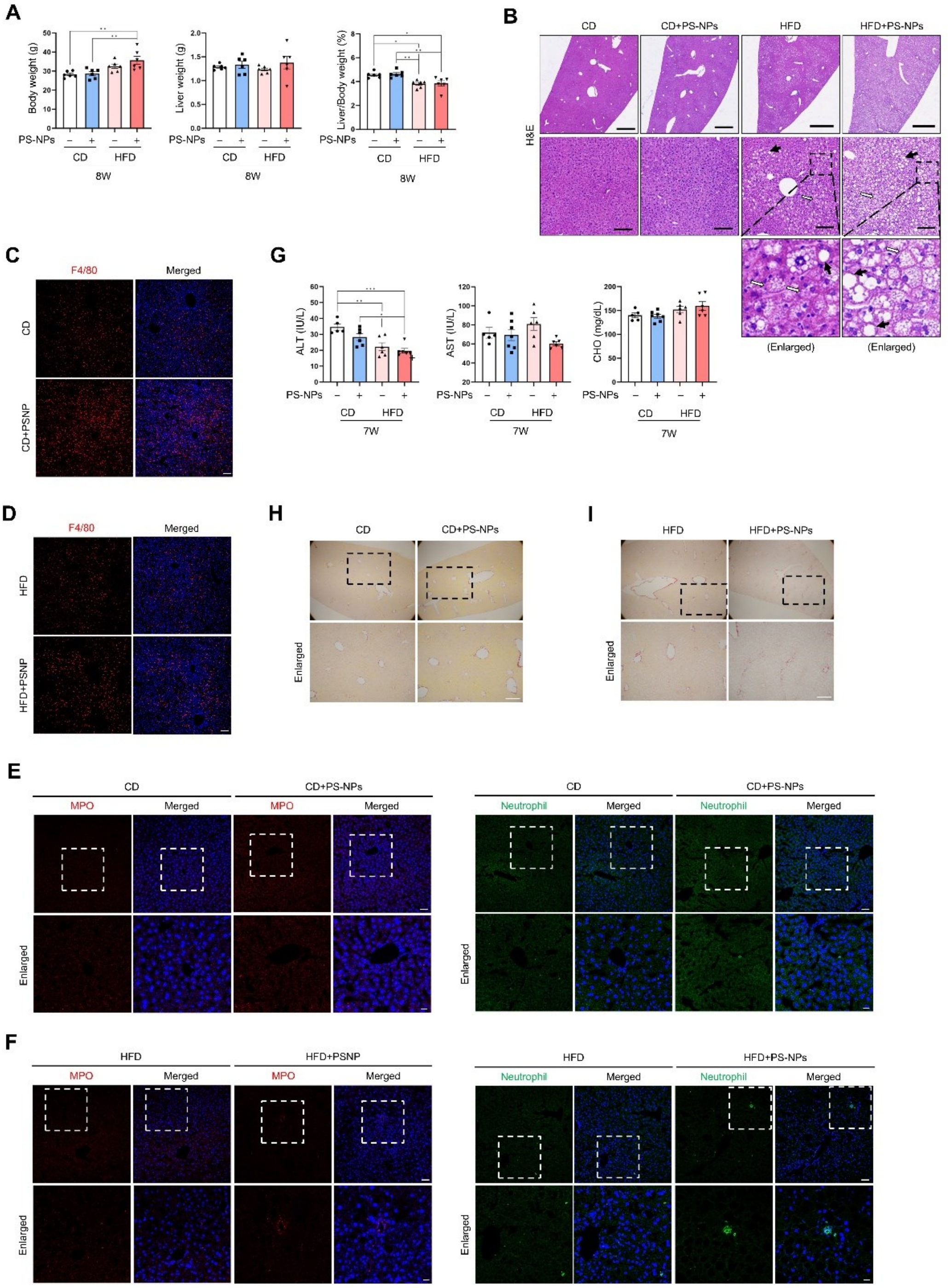

Figure S1. Animal experiments: 8- or 20-week feeding of PS-NPs and CD or HFD. (A) Body weight, liver weight, and liver-to-body weight ratio of mice exposed to 0 or 0.5 mg PS-NPs for 8 weeks. n = 4–6 in each group. (B) Representative images of H&E staining of liver from mice with indicated diet for 20-week (upper, × 40; lower, × 200; and enlarged). Macrovesicular (black arrow) and microvesicular (white arrow) fatty changes observed in the HFD and HFD+PS-NPs groups. Scale bar: upper = 600 μm, lower = 120 μm. (C) Representative images of F4/80 immunofluorescence staining of liver section in mice fed CD or CD+PS-NPs for 20-week. Scale bar: upper = 50 μm, lower = 20 μm. (D) Representative images of F4/80 immunofluorescence staining of liver section in mice fed HFD or HFD+PS-NPs for 20-week. Scale bar: upper = 50 μm, lower = 20 μm. (E) Representative images of MPO and neutrophil immunofluorescence staining of liver section in mice fed CD or CD+PS-NPs for 20-week. Scale bar: upper = 50 μm, lower = 20 μm. (F) Representative images of MPO and neutrophil immunofluorescence staining of liver section in mice fed HFD or HFD+PS-NPs for 20-week. Scale bar: upper = 50 μm, lower = 20 μm. (G) Alanine aminotransferase (ALT), aspartate aminotransferase (AST), and cholesterol (CHO) levels in serum of mice treated with or without PS-NPs and/or HFD for 7 weeks. (H) Representative images of Sirius Red staining of liver section in mice fed CD or CD+PS-NPs for 20-week. Scale bar: upper = 500 μm, lower = 20 μm. (I) Representative images of Sirius Red Staining of liver section in mice fed HFD or HFD+PS-NPs for 20-week. Scale bar: upper = 500 μm, lower = 20 μm. Error bars represent SEM. *$p < 0.05$, **$p < 0.01$, ***$p < 0.001$, ****$p < 0.0001$ vs. CD-PS-NPs, by ordinary one-way ANOVA and Tukey’s multiple comparison tests. CD; chow diet, HFD; high-fat diet.

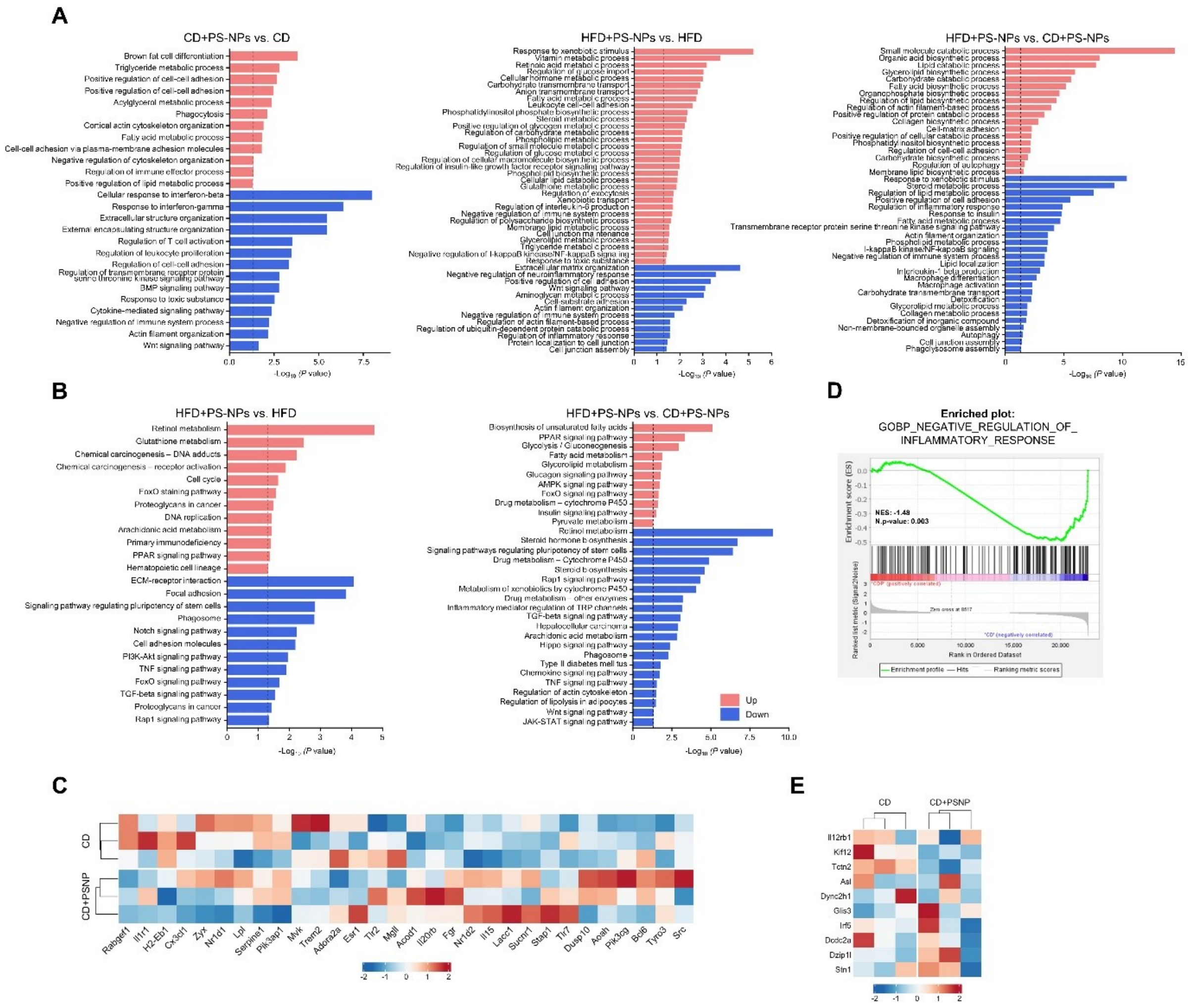
A
CD+PS-NPs vs. CD
Brown fat cell differentiation
Triglyceride metabolic process
Positive regulation of cell-cell adhesion
Positive regulation of cell-cell adhesion
Acylglycerol metabolic process
Phagocytosis
Cortical actin cytoskeleton organization
Fatty acid metabolic process
Cell-cell adhesion via plasma-membrane adhesion molecules
Negative regulation of cytoskeleton organization
Regulation of immune effector process
Positive regulation of lipid metabolic process
Cellular response to interferon-beta
Response to interferon-gamma
Extracellular structure organization
External encapsulating structure organization
Regulation of T cell activation
Regulation of leukocyte proliferation
Regulation of cell-cell adhesion
Regulation of transmembrane receptor protein serine threonine kinase signaling pathway
BMP signaling pathway
Response to toxic substance
Cytokine-mediated signaling pathway
Negative regulation of immune system process
Actin filament organization
Wnt signaling pathway
0.0 2.5 5.0 7.5
-Log10 (P value)
HFD+PS-NPs vs. HFD
Response to xenobiotic stimulus
Vitamin metabolic process
Retinoic acid metabolic process
Regulation of glucose import
Cellular hormone metabolic process
Carbohydrate transmembrane transport
Anion transmembrane transport
Fatty acid metabolic process
Leukocyte cell-cell adhesion
Phosphatidylinositol phosphate biosynthetic process
Steroid metabolic process
Positive regulation of glycogen metabolic process
Regulation of carbohydrate metabolic process
Phospholipid metabolic process
Regulation of small molecule metabolic process
Regulation of glucose metabolic process
Regulation of cellular macromolecule biosynthetic process
Regulation of insulin-like growth factor receptor signaling pathway
Phospholipid biosynthetic process
Cellular lipid catabolic process
Glutathione metabolic process
Regulation of exocytosis
Xenobiotic transport
Regulation of interleukin-6 production
Negative regulation of immune system process
Regulation of polysaccharide biosynthetic process
Membrane lipid metabolic process
Cell junction maintenance
Glycerolipid metabolic process
Triglyceride metabolic process
Negative regulation of I-kappaB kinase/NF-kappaB signaling
Response to toxic substance
Extracellular matrix organization
Negative regulation of neuroinflammatory response
Positive regulation of cell adhesion
Wnt signaling pathway
Aminoglycan metabolic process
Cell-substrate adhesion
Actin filament organization
Negative regulation of immune system process
Regulation of actin filament-based process
Regulation of ubiquitin-dependent protein catabolic process
Regulation of inflammatory response
Protein localization to cell junction
Cell junction assembly
0 1 2 3 4 5 6
-Log10 (P value)
HFD+PS-NPs vs. CD+PS-NPs
Small molecule catabolic process
Organic acid biosynthetic process
Lipid catabolic process
Glycerolipid biosynthetic process
Carbohydrate catabolic process
Fatty acid biosynthetic process
Organophosphate biosynthetic process
Regulation of lipid biosynthetic process
Regulation of actin filament-based process
Positive regulation of protein catabolic process
Collagen biosynthetic process
Cell-matrix adhesion
Positive regulation of cellular catabolic process
Phosphatidylinositol biosynthetic process
Regulation of cell-cell adhesion
Carbohydrate biosynthetic process
Regulation of autophagy
Membrane lipid biosynthetic process
Response to xenobiotic stimulus
Steroid metabolic process
Regulation of lipid metabolic process
Positive regulation of cell adhesion
Regulation of inflammatory response
Response to insulin
Fatty acid metabolic process
Transmembrane receptor protein serine threonine kinase signaling pathway
Actin filament organization
Phospholipid metabolic process
I-kappaB kinase/NF-kappaB signaling
Negative regulation of immune system process
Lipid localization
Interleukin-1 beta production
Macrophage differentiation
Macrophage activation
Carbohydrate transmembrane transport
Detoxification
Glycerolipid metabolic process
Collagen metabolic process
Detoxification of inorganic compound
Non-membrane-bounded organelle assembly
Autophagy
Cell junction assembly
Phagolysosome assembly
0 5 10 15
-Log10 (P value)
B
HFD+PS-NPs vs. HFD
Retinol metabolism
Glutathione metabolism
Chemical carcinogenesis – DNA adducts
Chemical carcinogenesis – receptor activation
Cell cycle
FoxO staining pathway
Proteoglycans in cancer
DNA replication
Arachidonic acid metabolism
Primary immunodeficiency
PPAR signaling pathway
Hematopoietic cell lineage
ECM-receptor interaction
Focal adhesion
Signaling pathway regulating pluripotency of stem cells
Phagosome
Notch signaling pathway
Cell adhesion molecules
PI3K-Akt signaling pathway
TNF signaling pathway
FoxO signaling pathway
TGF-beta signaling pathway
Proteoglycans in cancer
Rap1 signaling pathway
0 1 2 3 4 5
-Log10 (P value)
HFD+PS-NPs vs. CD+PS-NPs
Biosynthesis of unsaturated fatty acids
PPAR signaling pathway
Glycolysis / Gluconeogenesis
Fatty acid metabolism
Glycerolipid metabolism
Glucagon signaling pathway
AMPK signaling pathway
FoxO signaling pathway
Drug metabolism – cytochrome P450
Insulin signaling pathway
Pyruvate metabolism
Retinol metabolism
Steroid hormone biosynthesis
Signaling pathways regulating pluripotency of stem cells
Drug metabolism – Cytochrome P450
Steroid biosynthesis
Rap1 signaling pathway
Metabolism of xenobiotics by cytochrome P450
Drug metabolism – other enzymes
Inflammatory mediator regulation of TRP channels
TGF-beta signaling pathway
Hepatocellular carcinoma
Arachidonic acid metabolism
Hippo signaling pathway
Phagosome
Type II diabetes mellitus
Chemokine signaling pathway
TNF signaling pathway
Regulation of actin cytoskeleton
Regulation of lipolysis in adipocytes
Wnt signaling pathway
JAK-STAT signaling pathway
Up
Down
0 2.5 5.0 7.5 10.0
-Log10 (P value)
D
Enriched plot:
GOBP_NEGATIVE_REGULATION_OF_INFLAMMATORY_RESPONSE
Enrichment score (ES)
NES: -1.48
N.p-value: 0.003
'CDP' (positively correlated)
Zero cross at 8517
'CD' (negatively correlated)
Ranked list metric (Signal2Noise)
Rank in Ordered Dataset
Enrichment profile — Hits — Ranking metric scores
C
CD
CD+PSNP
Rabgef1 Il1r1 H2-Eb1 Cx3cl1 Zyx Nr1d1 Lpl Serpine1 Pik3ap1 Mvk Trem2 Adora2a Esr1 Tlr2 Mgll Acod1 Il20rb Fgr Nr1d2 Il15 Lacc1 Sucnr1 Stap1 Tlr7 Dusp10 Aoah Pik3cg Bcl6 Tyro3 Src
-2 -1 0 1 2
E
CD
CD+PSNP
Il12rb1
Kif12
Tctn2
Asl
Dync2h1
Glis3
Irf5
Dcdc2a
Dzip1l
Stn1
-2 -1 0 1 2

Figure S2. DEG analysis. Significantly enriched gene ontology biological process (GOBP) terms (A) and Kyoto Encyclopedia of Genes and Genomes (KEGG) pathway terms (B) of CD+PS-NPs vs. CD, HFD+PS-NPs vs. HFD, and HFD+PS-NPs vs. CD+PS-NPs groups. The dotted line represents the *P* value threshold of 0.05, with pink bars indicating upregulated pathways and blue bars indicating downregulated pathways. (C) Heatmap of the RNA-seq results showing differences in the expression of inflammatory genes in the liver of CD or CD+PS-NPs groups. The color scale ranges from - 2 (blue) to 2 (red). (D) Gene set enrichment analysis (GSEA) enrichment plot of inflammation-related genes in the liver of CD+PS-NPs vs. CD group. (E) Heatmap of the RNA-seq results showing differences in the expression of fibrogenic-related genes in the liver of CD or CD+PS-NPs groups. The color scale ranges from - 2 (blue) to 2 (red).

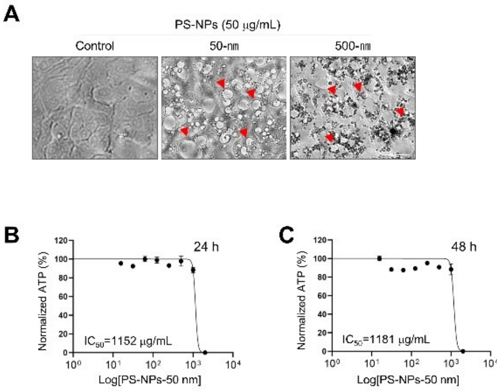
A
PS-NPs (50 μg/mL)
Control
50-nm
500-nm
B
24 h
Normalized ATP (%)
IC50=1152 μg/mL
Log[PS-NPs-50 nm]
C
48 h
Normalized ATP (%)
IC50=1181 μg/mL
Log[PS-NPs-50 nm]

Figure S3. Cytotoxicity of PS-NPs in HepaRG cells. (A) Comparison of vacuoles (red arrowhead) induced by 50 nm and 500 nm PS-NPs. Scale bar: 50 μm. Evaluation of HepaRG cell viability after PS-NP exposure involved treating the cells with serial twofold dilutions of PS-NPs, ranging from 0 μg/mL to 2,000 μg/mL. After 24 h (B) or 48 h (C), cell viability was assessed using ATP content as a control. The graphs depict the half-maximal inhibitory concentration (IC_{50}) of PS-NPs.

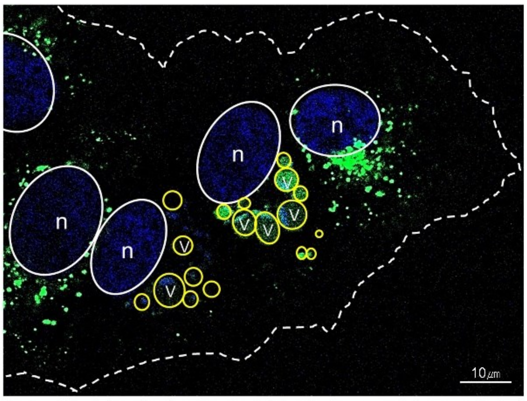
n
n
n
n
n
V
V
V
V
V
V
10 μm

Figure S4. Internalization analysis of PS-NPs using fluorescein-conjugated dextran and vacuole formation. Representative image of HepaRG cells after incubated with 50 µg/mL PS-NPs and 0.1 mg/mL fluorescein-conjugated dextran (green) for 24 h and 30 min, respectively. The image was captured using a confocal microscope with ×100 objective magnification is shown. Scale bar: 10 µm. n; nucleus, v; vacuoles.

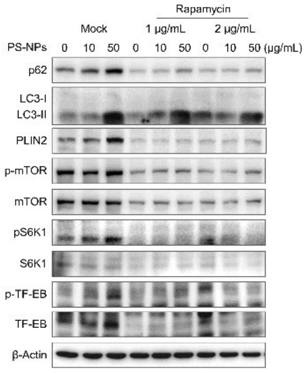
Rapamycin
Mock
1 μg/mL
2 μg/mL
PS-NPs
0 10 50 0 10 50 0 10 50 (μg/mL)
p62
LC3-I
LC3-II
PLIN2
p-mTOR
mTOR
pS6K1
S6K1
p-TF-EB
TF-EB
β-Actin

Figure S5. Rapamycin recovered autophagy disruption induced by PS-NPs. (A) HepaRG cells were pretreated with 1 or 2 μg/mL rapamycin, an mTOR inhibitor and autophagy enhancer, for 3 h and subsequently exposed to PS-NPs at the indicated concentrations.

SUPPLEMENTARY TABLES

Table S1. List of reagents used in the study

Reagents	Company	Catalog No.	Stock Cont.	Working Cont.
Dimethyl sulfoxide	SantaCruz	sc-358801		
Bafilomycin A1	InvivoGen	tlrl-baf1	100 μM	10 nM
3-Methyladenine	Sigma	M9281	100 mM	5 mM
5-(N-ethyl-N-isopropyl) amiloride	Sigma	A3085	100 mM	3 μM
Chlorpromazine hydrochloride	Sigma	31679	200 mM	5 μM
Cytochalasin B from *Drechslera dematioidea*	Sigma	C6762	10 mM	1 μM
Genistein	Sigma	G6649	20 mM	100 μM
Rapamycin	Sigma	37094	1 mg/mL	2 μg/mL
Sodium oleate	Sigma	O7501	5 mM	0.5 mM
BODIPY™ 493/503	Invitrogen	D3922	3.8 mM	5 μM
Nile Red	Invitrogen	N1142	0.5 mg/mL	5 μg/mL
Dextran	Invitrogen	D1845	25 mg/mL	50 or 100 μg/mL
ER-Tracker™ Red	Invitrogen	E34250	1 mM	1 μM
MitoTracker™ Deep Red FM	Invitrogen	M22426	1 mM	1 μM
LysoTracker™ Red DND-99	Invitrogen	L7528	1 mM	1 μM
LysoSensor™ Yellow/Blue DND-160	Invitrogen	L7545	1 mM	1 μM
Hoechst 33258	Invitrogen	H3569	10 mg/mL	10 μg/mL
Oil Red O	Sigma	O1391	0.5%	
Crystal violet	Sigma	C0775	0.5%	0.5%
Acetic acid, glacial	Sigma	A6283	99%	
Ethanol	Merck	1.00983.1011	99.9%	
2-Propanol	Merck	1.09634.1011	99.8%	
Methanol	Merck	1.06009.1011	99.9%	
Xylene	Junsei	25165S0480	85%	

Mayer's Hematoxylin	Dako	S3309
Eosin	Dako	CS701
Triglyceride (TG)	Beckman-Coulter	OSR61118
Alanine aminotransferase (ALT)	Beckman-Coulter	OSR6107
Aspartate aminotransferase (AST)	Beckman-Coulter	OSR6109
Cholesterol (CHO)	Beckman-Coulter	OSR6116
Triglyceride assay kit	Abcam	ab65336
Cathepsin B activity fluorescence-based assay kit	Abcam	ab65300
Picro-Sirius Red	Abcam	Ab246832

No., Numbers
Cont., Concentrations

Table S2. List of primary antibodies used in this study

Antibody	Host species	Source	Catalog No.
β-actin	Mouse	Enogene	E12-041
C/EBPα	Mouse	Santa Cruz	sc-365318
C/EBPβ	Mouse	Santa Cruz	sc-7962
Cathepsin D	Mouse	Abcam	ab6313
EEA1	Rabbit	CST	3288
LAMP1	Rabbit	CST	9091
LAMP1	Mouse	Santa Cruz	sc-20011
LAMP2	Mouse	Santa Cruz	sc-18822
LC3A/B	Rabbit	CST	4108
LC3B	Mouse	Santa Cruz	sc-376404
LGP85	Mouse	Santa Cruz	sc-55570
mToR	Rabbit	CST	2983
Myeloperoxidase	Rabbit	Abcam	ab208670
Neutrophil	Rat	Abcam	ab2557
PLIN2	Rabbit	Abcam	ab108323
p38α	Mouse	Santa Cruz	sc-81621
p62	Rabbit	CST	8025
PPARα	Mouse	Santa Cruz	sc-398394
PPARγ	Rabbit	CST	2435
RAB7	Rabbit	CST	9367
S6K	Rabbit	CST	9202
TFEB	Rabbit	CST	4240
MYC	Mouse	Santa Cruz	sc-40
Phospho-mToR	Rabbit	CST	5536
Phospho-p70S6K	Mouse	CST	9206
Phospho-TFEB	Rabbit	CST	37681

CST, Cell Signaling Technology

Table S3. List of secondary antibodies used in this study

Antibody	Company	Catalog No.
Goat anti-Mouse IgG (H+L) Cross-Adsorbed Secondary Antibody, Alexa Fluor™ 488	Invitrogen	A11001
Goat anti-Rabbit IgG (H+L) Cross-Adsorbed Secondary Antibody, Alexa Fluor™ 488	Invitrogen	A11008
Goat anti-Mouse IgG (H+L) Highly Cross-Adsorbed Secondary Antibody, Alexa Fluor™ 594	Invitrogen	A11032
Goat anti-Rabbit IgG (H+L) Cross-Adsorbed Secondary Antibody, Alexa Fluor™ 594	Invitrogen	A11012
Goat anti-Rat IgG (H+L) Cross-Adsorbed Secondary Antibody, Alexa Fluor ™ 488	Invitrogen	A11006
Goat Anti-Mouse IgG -HRP	AbFrontier	LF-SA8001
Goat Anti-Rabbit IgG-HRP	AbFrontier	LF-SA8002

Table S4. List of primer sequences

Symbol	Forward primer (5'→3')	Reverse primer (5'→3')
Albumin (*ALB*)	AGAATGCGCTATTAGTTCGT	ACTTACTGGCGTTTTCTCAT
CD36	AGATGCAGCCTCATTTCCAC	GCCTTGGATGGAAGAACAAA
CEBPA	GTGGACAAGAACAGCAACGA	GTCATTGTCACTGGTCAGCTC
CEBPB	CGCTTACCTCGGCTACCAG	TTGTACTCGTCGCTGTGCTT
CYP3A4	TTTTGTCCTACCATAAGGGC	CATAAATCCCACTGGACCAA
FABP1	GCAGAGCCAGGAAAACTTTG	TCTCCCCTGTCATTGTCTCC
HO-1	CAGTGCCACCAAGTTCAAGC	TTGAGCAGGAACGCAGTCTT
HNF4a	GCTCGGAGCCACCAAGAGAT	CGTATGGACACCCGGCTCAT
IL6	GGTACATCCTCGACGGCATCT	GTGCCTCTTTGCTGCTTTCAC
IL6	GGTACATCCTCGACGGCATCT	GTGCCTCTTTGCTGCTTTCAC
LC3B	GTCAGCGTCTCCACACCAA	TTTCATCCCGAACGTCTCCT
P62	AGGCGCACTACCGCGAT	CGTCACTGGAAAAGGCAACC
MYC	GAGGAGGAACGAGCTAAAAC	GAGTTCCGTAGCTGTTCAAG
PGC-1A	AAGGATGCGCTCTCGTTCAA	AAGGGAGAATTTCGGTGCGT
PLIN2	GCTGAGCACATTGAGTCACG	TGGTACACCTTGGATGTTGG
PPARG	GATGTCTCATAATGCCATCAGGT	TCAGCGGACTCTGGATTCAG
RPL13A	CATAGGAAGCTGGGAGCAAG	GCCCTCCAATCAGTCTTCTG
